# Work-family life courses and BMI trajectories in three British birth cohorts

**DOI:** 10.1038/ijo.2016.197

**Published:** 2016-11-29

**Authors:** R E Lacey, A Sacker, S Bell, M Kumari, D Worts, P McDonough, D Kuh, A McMunn

**Affiliations:** 1 Department Epidemiology and Public Health, University College London, London, UK; 2 Institute for Social and Economic Research, University of Essex, Colchester, UK; 3 Dalla Lana School of Public Health, University of Toronto, Toronto, Ontario, Canada; 4 MRC Unit for Lifelong Health and Ageing at UCL, London,UK

## Abstract

**BACKGROUND/OBJECTIVES:**

Combining work and family responsibilities has previously been associated with improved health in mid-life, yet little is known about how these associations change over time (both biographical and historical) and whether this extends to body mass index (BMI) trajectories for British men and women. The purpose of this study was to investigate relationships between work-family life courses and BMI trajectories across adulthood (16–42 years) for men and women in three British birth cohorts.

**SUBJECTS/METHODS:**

Multiply imputed data from three nationally representative British birth cohorts were used—the MRC National Survey of Health and Development (NSHD; 1946 birth cohort, *n*=3012), the National Child Development Study (NCDS; 1958 birth cohort, *n*=9614) and the British Cohort Study (BCS; 1970 birth cohort, *n*=8140). A typology of work-family life course types was developed using multi-channel sequence analysis, linking annual information on work, partnerships and parenthood from 16 to 42 years. Work-family life courses were related to BMI trajectories using multi-level growth models. Analyses adjusted for indicators of prior health, birthweight, child BMI, educational attainment and socioeconomic position across the life course, and were stratified by gender and cohort.

**RESULTS:**

Work-family life courses characterised by earlier transitions to parenthood and weaker long-term links to employment were associated with greater increases in BMI across adulthood. Some of these differences, particularly for work-family groups, which are becoming increasingly non-normative, became more pronounced across cohorts (for example, increases in BMI between 16 and 42 years in long-term homemaking women: NSHD: 4.35 kg m^–2^, 95% confidence interval (CI): 3.44, 5.26; NCDS: 5.53 kg m^–^^2^, 95% CI: 5.18, 5.88; BCS: 6.69 kg m^–^^2^, 95% CI: 6.36, 7.02).

**CONCLUSIONS:**

Becoming a parent earlier and weaker long-term ties to employment are associated with greater increases in BMI across adulthood in British men and women.

## Introduction

With a few exceptions,^1, 2^ combining paid work and family responsibilities has been shown to benefit health in mid-life.^3, 4, 5, 6, 7, 8, 9^ Much of this work has focused on women, whose working lives are typically more entwined with caring responsibilities.^10, 11^ For instance, previous analyses of the Medical Research Council (MRC) National Survey of Health and Development (NSHD, the 1946 British birth cohort) showed that women with relatively strong ties to employment and marriage had better health than women who spent long periods of time out of work looking after the home and family.^3^ The timing of key life course transitions, such as becoming a parent, are also likely to be important for subsequent health. Indeed research has shown that early parenthood is associated with heightened risk of mortality^12^ and cardiovascular disease.^13^ This may be particularly true for women—and all the more so when combined with weak ties to paid work and partnerships.^7, 8^

Work-family life courses potentially affect health through biological and behavioural stress mechanisms.^14^ For example, weak ties to paid work and/or to marriage have been linked to increased physiological stress, as have earlier transitions to parenthood.^7, 8, 15, 16, 17^ Employment is important for enabling access to material resources and extended social networks, and also encourages wider participation in society.^18^ Early transitions to parenthood can interrupt career and educational opportunities, and also reduce the social control of health behaviours, such as physical activity.^19^ Stress exposure may be related to physical health outcomes, either indirectly through risky health behaviours or directly through long-term alterations in physiological stress responses.^19, 20, 21^ Therefore, it is plausible that work-family life courses may be related to differences in body mass index (BMI). Identifying any such associations is important, given that raised BMI has been associated with elevated risks of developing many later health conditions, such as cardiovascular disease^22^ and type 2 diabetes.^23^

Prior research investigating the health sequelae of combining work and family life for health has been limited in several ways. First, men are frequently excluded from analyses, often under the (possibly mistaken) assumption that combining work and family matters less for men. Second, studies of social roles and health have typically been limited to data collected at only one or two time points. This fails to capture changes in work and family life courses over time, and precludes the possibility of addressing questions of causal direction between work, family and health. Third, with a few exceptions,^1, 7, 8, 24^ there has been a reliance on subjective measures of health, which may be biased by participants' satisfaction with their work or family experiences or unmeasured potential confounders such as personality attributes. Fourth, few studies have adjusted for potential selection on the basis of early-life health and socioeconomic factors thought to influence both work and family opportunities and subsequent health; hence previous estimates may be biased in important ways. Finally, few studies have examined whether associations of work and family with health have changed over historical time. There is a societal expectation that key life course experiences, such as parenthood, paid work and partnerships, are ordered and timed in certain ways.^25^ Deviations from this in the form of non-normative life courses, such as earlier parenthood, unemployment or partnership dissolution, may result in increased stress and consequently poorer health.^20^ Moreover, norms concerning work-family life courses have changed over time, with later transitions to parenthood, increasing cohabitation, lower marriage rates and stronger ties to paid work for women, increasingly becoming the norm in recent decades.^26^ Limited evidence suggests that as the timing of key life course transitions (for example, entry into parenthood) shifts, formerly normative transitions may become progressively more problematic for health.^27^

The aim of this study was to test relationships between combined work-family life courses and BMI trajectories for men and women in three British birth cohorts. Work-family life courses characterised by weaker links to employment and earlier transitions to partnerships and parenthood were anticipated to be associated with steeper increases in BMI across the adult life course. It was also hypothesised that BMI increases among those with weak labour force attachment and early parenthood would become more pronounced over historical time (that is, across cohorts), reflecting the increasingly non-normative nature of these work-family life courses. Given the previous focus on the health of women in relation to work-family life courses, we also investigated whether their associations with BMI trajectories varies significantly by gender.

## Subjects and methods

### Participants

Data from three nationally representative British birth cohorts were used. The oldest study, the NSHD, is a stratified random sample of all births in 1 week of 1946 in Great Britain.^28^ Study members were babies born to married women with husbands in non-manual or agricultural work, and one-quarter of babies born to women with husbands in manual work (*n*=5362). Sampling weights were included in analyses to allow for the unequal selection probability. Participants were interviewed on numerous occasions, including 15 times before age 25, and at ages 26, 31, 36, 43, 54 and 60–64 years. Bona fide researchers can apply to access NSHD data via a standard application procedure (further details available at: http://www.nshd.mrc.ac.uk/data.aspx).

The National Child Development Study (NCDS, also known as the 1958 birth cohort) aimed to recruit all babies born during a single week of 1958 (achieved sample= 17 415, 98.2% of target).^29^ Similar to the NSHD, participants have been interviewed on many occasions: birth, 7, 11, 16, 23, 33, 42, 44/45, 46, 50 and 55 years. The British Cohort Study (BCS, also known as the 1970 birth cohort) sought to recruit all babies born during 1 week of 1970 (achieved sample= 16 571, 95.9% of target).^30^ Participants have been surveyed at 5, 10, 16, 26, 30, 34, 38 and 42 years. NCDS and BCS data are available from the UK Data Service (www.ukdataservice.ac.uk). All three cohorts have collected information on social, developmental, health and economic aspects of participants' lives. All available waves in the three studies, from adolescence to age 42 (age 43 in the NSHD), were used, reflecting age at the most recent BCS survey. Response rates for the age 42/43 surveys were: NSHD 87.0% (3262 of target sample of 3749),^28^ NCDS 70.3% (11 419 of target sample of 16 240)^29^ and BCS 84.5% (9842 of target sample of 11 654).^31^ Informed consent and ethics committee approval was obtained for all three cohorts.

### Measures

#### Work-family life courses

Annual information on work, partnership and parenthood status was derived for ages 16–42 years. Work status was full-time employment, part-time (⩽30 h per week) employment, looking after the home and family or other not employed (unemployed, in education or training, sick, or not working for another reason). Partnership status was categorised as married, cohabiting or not living with a partner. Parental status was defined as no children in the household or youngest child >16 years, youngest child in the household <5 years, or youngest child in the household aged 5–16 years. These three domains were collapsed to produce 26 annual work-family state variables (one for each year between ages 16 and 42 years), each with 36 categories (4 employment × 3 partnership × 3 parental).

#### Body mass index

Height and weight were available at five ages in the NSHD (15, 20 (self-reported), 26 (self-reported), 36 and 43 years), four ages in the NCDS (16, 23, 33, 42 (self-reported) years) and five ages in the BCS (16, 26, 30, 34 and 42 years, all self-reported except for age 16). BMI was calculated using the standard formula of weight (kg) height (m)^–^^2^.

#### Covariates

Covariates included birthweight (kg), early-life health, and childhood and adult socioeconomic position. Comparable information on internalising and externalising disorders existed in all three studies. The Rutter behavioural scales were measured in the NCDS and BCS and a pre-cursor to the Rutter behaviour scale was available in the NSHD. Factor analysis was used to derive internalising and externalising disorders and scores were then categorised based on recognised percentile cut-offs.^32^ For internalising disorders, these cut-point scores were: 0–50% (absent), 51–87% (mild) and ⩾88% (severe); and for externalising disorders, they were: 0–75% (absent), 75–93% (mild) and ⩾94% (severe). The existence of health problems was reported in medical examinations at age 16 in the NCDS and BCS, and age 15 in the NSHD. We also included information on hospital admissions from ages 11 in the NSHD (The NSHD hospital admission data referred to admissions for >28 days, but data were only available in the NCDS and BCS on whether the child had been admitted or not, regardless of length of stay.) and NCDS, and age 10 in the BCS. A measure of childhood BMI from age 11 in the NSHD and NCDS, and age 10 in the BCS, was also incorporated.

Indicators of life course socioeconomic position included father's social class (Registrar General's Social Class (RGSC) schema) at age 4 in the NSHD, age 7 in the NCDS and age 5 in the BCS categorised as: professional (I), managerial and technical (II), skilled non-manual (IIINM), unskilled non-manual (IIIM), semi-skilled manual (IV) or unskilled manual (V). Where information was missing at these ages, we used data on father's social class from subsequent childhood sweeps. Adult socioeconomic position was indicated by the highest RGSC in the household (where available (Own social class was used in the following surveys: NSHD—age 20 and 26, BCS—age 26 and 34. The highest social class of the cohort member and their partner was used at all other ages.)) at each adult survey between ages 15/16 and 42/43, taking the same categories as above. Educational attainment was coded as the highest qualification achieved by age 26 in the NSHD and BCS, and age 23 in the NCDS (no qualifications, Certificate of Secondary Education (CSE)/Ordinary-Level (O-level), Advanced-level (A-level) or higher qualification/degree). We also included information on parental interest in the child's education as rated by the child's teacher (little or no interest, average interest, or high interest) and reading comprehension scores, both from age 11 in the NSHD and NCDS, and age 10 in the BCS.

### Statistical analysis

#### Multi-channel sequence analysis

Multi-channel sequence analysis was used to group individuals' work-family life courses in each cohort using the combined work-family variables described above. Sequence analysis matched each participant's work-family sequence to its closest pre-defined ‘ideal-type' reference sequence. The set of ‘ideal-type' sequences reflected the most common work-family combinations and were based on previous familiarity with these cohorts. More details on the sequence analysis method can be found in McMunn *et al.*^26^ Table 1 describes the 12 ‘ideal types' and gives distributions for the ensuing work-family trajectory groups in all three cohorts. Four work-family types—‘Later family, work break' ‘Early family, work break' ‘Part-time work, early family' and ‘No paid work, early family'—contained too few men in any of the three cohorts to produce reliable estimates. These groups are therefore only considered in the analyses of women.

#### Multiple imputation

Missing data on work, partnerships and parental status was imputed Halpin's^33^ method, which overcomes problems of collinearity and imprecise estimation for missing sequence information. Twenty imputed data sets were created, resulting in complete work-family information for 3012 NSHD participants, 9616 NCDS participants and 8158 BCS participants. Further information on the application of this method can be found in McMunn *et al.*^26^ Subsequently, missing information on all covariates was imputed using multiple imputation by chained equations for those with at least one observed BMI measure (NSHD: *n*=3012, NCDS: *n*=9608, BCS: *n*= 8140—the analytic samples for this study).

#### Growth curve modelling

The relationship between work-family life courses and longitudinal change in BMI was tested using multi-level growth models (allowing for random intercepts and slopes) using a maximum likelihood algorithm. Models included age, age-squared, work-family type and a work-family type-by-age interaction. Models additionally included the following time-invariant variables: birthweight, early-life health, childhood BMI, educational attainment and social class (time-varying). The intercept was set at age 43 in the NSHD and age 42 in the NCDS and BCS. Regression coefficients were converted to predicted mean BMIs at ages 16 and 42 years for each work-family group in order to aid interpretation. Additional models (data not shown) found statistically significant gender–age interactions in all three cohorts and gender work-family type interactions significant in the NCDS; therefore all analyses were stratified by cohort and gender. Analyses were conducted in Stata version 13,^34^ and using the seqcomp plug-in for the sequence analysis.^35^

## Results

The distribution of work-family life courses of men and women by cohort is shown in Table 1. Work-family life courses, which would now be perceived as non-normative, that is, characterised by weaker ties to paid work for women and early parenthood, become less prevalent with each successive cohort. The mean observed BMIs of men and women in each cohort are shown in Supplementary Figure 1. Average BMIs increased for both genders over the life course and across cohorts, and aside from age 16, men's BMIs were higher than women's in adulthood.

### Work-family life courses and BMI trajectories for men

The predicted mean BMIs (adjusted for all covariates of interest) at ages 16 and 42 years by work-family type for men are presented in Table 2 (the regression coefficients, on which these are based, are shown in Supplementary Table 1). In the NSHD, a very small group of men who made early transitions to parenthood (‘Teen parents') experienced greater average increases in BMI (mean increase: 6.46 kg m^–^^2^, 95% CI: 5.98, 6.93) than men with other work-family combinations. Mid-life BMI was also higher among men who experienced a divorce (‘Work, divorced parent' group; mean increase: 5.86 kg m^–^^2^, 95% CI: 5.16, 6.58).

As in the NSHD, NCDS men in the ‘Teen parent' type experienced significantly more BMI growth than other men. Men who made earlier transitions to family life (‘Work, early family') also had a large increase in BMI across adulthood. In the BCS, mean BMI for men in childless work-family groups or those characterised by later transitions to parenthood increased significantly less than for men in work-family life courses characterised by early transitions to parenthood. An exception were men in the ‘Unstable work, no family' group who also had larger BMI increases (mean increase: 7.02 kg m^–2^, 95% CI: 6.85, 7.19).

It is worth noting that average BMIs for men at age 42 in all of the work-family groups in the three cohorts were higher than the WHO threshold for overweight. The only exceptions are NSHD men in the ‘Work, later family' and ‘Work, no family' groups. In the BCS, men in the ‘Work, marriage, non-parent' and ‘Unstable work, no family' groups were very nearly at the threshold for obesity by the age of 42, on average.

### Work-family life courses and BMI trajectories for women

**Figure 1:**
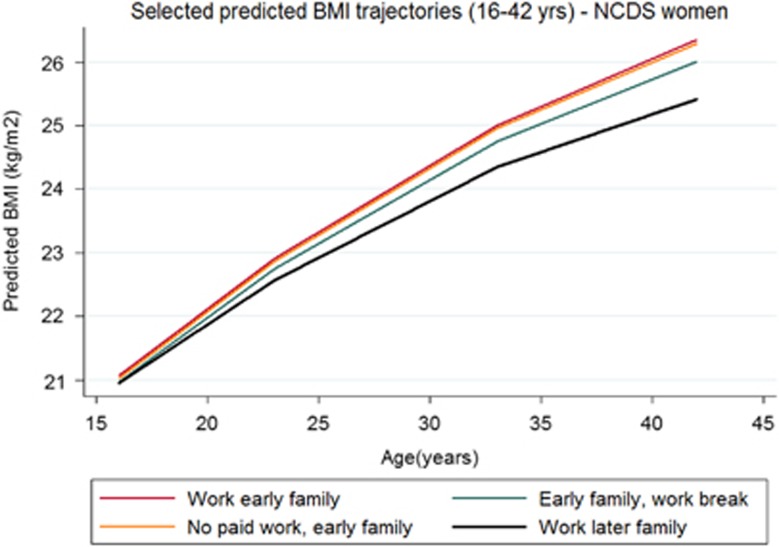
Selected predicted BMI trajectories (16–42 years)—NCDS women.

**Figure 2:**
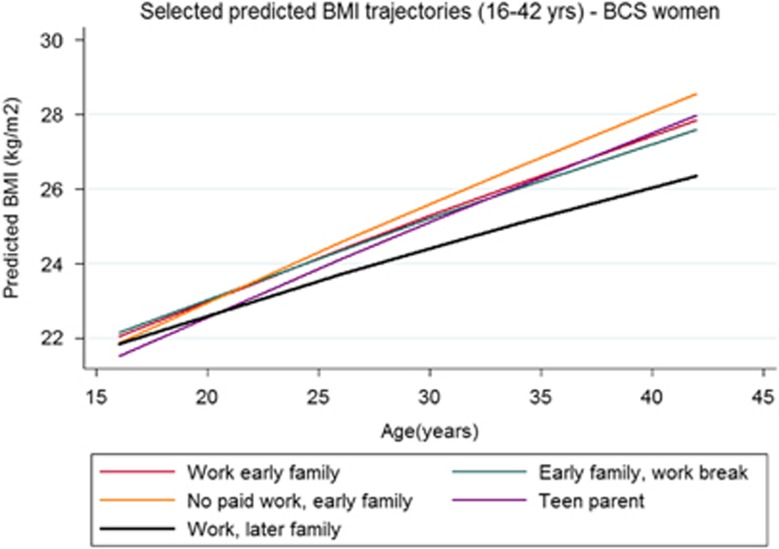
Selected predicted BMI trajectories (16–42 years)—BCS women.

Average BMI increases did not differ by work-family trajectory type for women in the NSHD (Table 3, regression coefficients presented in Supplementary Table 2). However, the BMI of NCDS women in work-family groups characterised by later transitions to parenthood increased significantly less than that of women who made early transitions to parenthood (Figure 1). In the BCS, long-term homemakers (‘No paid work, early family') had the largest increase in BMI; in fact, the gap between the BMI for this group and that for others with paid work grew across the three cohorts as this became an increasingly non-normative group (NSHD: 4.72 kg m^–^^2^, 95% CI: 4.00, 5.45; NCDS: 5.31 kg m^–^^2^, 95% CI: 4.97, 5.66; BCS: 6.88 kg m^–^^2^, 95% CI: 6.51, 7.26; *P*-value for cohort change ⩽0.001; see Figure 2 for difference for BCS women). Women in the ‘Teen parent' group experienced the second largest increase in BMI in the BCS cohort.

Average BMIs for women in all of the work-family groups in the BCS were higher than the WHO threshold for overweight, with long-term homemakers (‘No paid work, early family'), working mothers who had their children early (‘Work, early family) and ‘Teen parents' very nearly at the threshold for obesity by the age of 42, on average. NSHD women who were not married, not parents or not in paid work were also almost at the threshold for obesity by the age of 42.

The distribution of all covariates by cohort and work-family type is shown in Supplementary Table 3 for men and Supplementary Table 4 for women.

## Discussion

Using prospective data from three British birth cohorts, we have shown that work-family life courses were related to differential growth in BMI across adulthood. More specifically, work-family life courses characterised by earlier transitions to parenthood and weaker ties to paid work were associated with larger increases in BMI over the adult life course, even after accounting for differences in birthweight, child BMI, prior health, educational attainment and socioeconomic position. In some cases, and particularly for women who were long-term homemakers, this difference became more pronounced over historical time.

The findings of this study are consistent with previous work showing that early parenthood may have long-lasting implications for health.^7, 8, 12, 13, 27, 36, 37^ We extend this to include BMI trajectories across adulthood in three British birth cohorts. Earlier transitions to parenthood may disrupt educational and career trajectories^38, 39, 40^ resulting in increased stress and consequently increased growth in BMI. Earlier transitions to parenthood may also be linked to increasing obesity through unhealthy behaviours. For example, later transitions to parenthood have been related to increased social control of risky health behaviours, and higher participation in physical activity in later life.^19^ Similarly, prior studies have proposed that parents with preschool children may be less likely than parents with older children and those without children to have time to exercise.^41^ When parenthood commences earlier in life, unhealthy behaviours (such as absence of physical activity) are established and continue throughout adulthood.^42^ In our study, we adjusted for socioeconomic position from childhood onwards, therefore our findings suggest that the association between early parenthood and subsequent BMI operates over and above socioeconomic circumstances, at least as captured by occupational class.

Our study also suggests that the combination of earlier transitions to becoming a parent and weaker links with paid work (for example, long-term homemakers) may be particularly problematic for BMI. Our findings are consistent with previous work showing that women who were long-term homemakers had a greater increase in BMI across adulthood, compared with women who occupied multiple social roles.^3^ Similarly, American research has shown that women making non-normative (early) transitions to parenthood had lower psychological well-being in late mid-life.^27^ The addition of two more recent cohorts of women suggests that differences in BMI between homemakers and others may continue to grow over time as women increasingly opt out of this work-family pattern.

Our findings further indicate that non-normative social roles may also be associated with poorer health among men. In the BCS, becoming a parent in their teens was associated with even more growth in BMI among men than among women; and this trajectory type put men at particular risk in every cohort. In addition, men in the ‘Work, divorced parent' group had greater increases in BMI in the NSHD. This was also the case for men in the ‘Unstable work, no family' in the BCS, suggesting that weak links with paid work, partnerships and family may be particularly disadvantageous for men. There is much evidence to show that marriage is especially beneficial for the health of men and, conversely, that divorce may have greater negative health impacts for men than women.^43, 44, 45^

Our findings must be considered in the context of several limitations. First, we were not able to include experiential aspects of work and family life, such as quality and satisfaction, which may have partly explained some of the associations seen here. There are also likely to have been important changes to the physical activity levels of working lives across cohorts, with members of the BCS being more likely to be in sedentary, non-manual work,^46^ possibly in combination with increasing reliance on vehicular transport to work. Second, some of the BMI measures, especially for the BCS, were self-reported rather than objectively assessed. Despite this, self-reported BMI was found to correlate highly with preceding or subsequently measured BMI (for example, in the NCDS the correlation between measured BMI at age 33 and self-reported BMI at age 42 was 0.753). A third limitation is that our sample was restricted to those with at least one observed BMI measure. Longitudinal birth cohort studies suffer from differential attrition, meaning that those who were more disadvantaged or less healthy are likely to be underrepresented in this study.^47^ If so, this is likely to have resulted in underestimates of associations seen here. However, maximum likelihood estimation allows for incorporation of participants without all BMI observations in analyses. Finally, the enormity of the data included in our sequence analysis of work-family life courses necessitated that we combine those out of paid work because of unemployment, full-time education and sickness into one group; therefore, it is not possible to disentangle unemployment from sickness effects in our ‘Unstable work, no family group'.

Limitations aside, our study has a number of strengths. The use of multi-channel sequence analysis allowed us to simultaneously consider work, partnerships and parenthood across the adult years, and to be one of the first studies to do so in relation to health. In addition, our use of the multiple measures of BMI available in the surveys allowed for more reliable estimation of BMI trajectories. Missing information was accounted for using multiple imputation techniques, therefore reducing bias associated with attrition and non-response. We also utilised prospectively collected data on both men and women in three nationally representative British birth cohorts. By comparing results across three cohorts born in 1946, 1958 and 1970, we explored work-family life course patterns and BMI trajectories in differing historical contexts. For example, these three cohorts experienced differences in the transition from school to work; 1946 study members would have experienced high employment rates but 1958 study members would have experienced higher rates of unemployment throughout the 1970s, although most would have found employment immediately leaving full-time education. In contrast, members of the 1970 cohort would have entered the labour market in the mid-1980s when unemployment rates were very high, particularly among young people.^46^ With respect to partnerships and parenthood, societal rates of cohabitation and divorce, as well as increasing parenthood outside of marriage or long-term partnerships, increased with each successive cohort.^48^ This changing historical context will have had implications for the work and family opportunities for members of the three birth cohorts. In our study, cross-cohort comparison suggests the strength of social norms in the social patterning of BMI across cohort. For example, for women, early parenthood and long periods of full-time homemaking were linked with greater increases in BMI in the most recent cohort of women for whom these patterns are no longer normative. This was not the case for the earliest cohort of women who were more likely to be homemakers. Finally, this study has included both genders in its investigation of work-family life courses and BMI and shown that work-family life courses are influential for the BMI trajectories of men as well as women.

In conclusion, our study suggests that earlier transitions to becoming a parent and weaker long-term ties to paid work are associated with increased growth in BMI across adulthood in three British birth cohorts. Further research is required to investigate the life course mechanisms involved.

## Figures and Tables

**Table 1:** Distribution of work-family types in the 1946, 1958 and 1970 British birth cohorts by gender

*Work-family type*	*Description of ‘ideal type'*	*NSHD (1946 cohort)*		*NCDS (1958 cohort)*		*BCS (1970 cohort)*	
		*Men %*,^a^ n*=1487*^b^	*Women %,* n*=1525*^b^	*Men %,* n*=4682*^b^	*Women %,* n*=4926*^b^	*Men %,* n*=3804*^b^	*Women %,* n*=4336*^b^
Work, later family	Continuous full-time employment; cohabiting mid-20s, married from late 20s; children from early 30s	28.7	3.0	33.9	8.6	29.7	11.4
Work, early family	Continuous full-time employment; married and children from early 20s	43.0	13.8	31.8	12.1	14.6	6.5
Work, marriage, non-parent	Continuous full-time employment; married from early 20s; no children	9.9	6.6	7.8	8.8	9.2	8.9
Work, no family	Continuous full-time employment; no partner or children	12.5	6.5	12.8	9.6	25.8	18.7
Work divorced parent	Continuous full-time employment; married from early 20s-late 30s, single from late 30s; children from early 20s	1.3	0.5	4.5	2.8	3.1	3.7
Teen parent	Homemaker until mid-20s; employed full-time from mid-20s; married from early 30s; children from late teens	0.4	2.5	0.8	1.3	0.5	3.0
Work, cohabitation, later parent	Continuous full-time employment; cohabiting from mid-20s; children from early 30s	2.0	0.9	6.8	4.9	13.3	10.9
Unstable work, no family	Working intermittently; no partner or children	1.5	0.9	1.1	0.6	2.8	2.0
Later family, work break^c^	Employed full-time until late 20s; homemaking from early 30s; married from mid-20s; children from early 30s	0.4	14.1	0.2	13.1	0.5	12.3
Early family, work break c	Employed full-time until early 20s, homemaking from early–late 20s; employed part-time from early 30s; marriage and children from early 20s	0	17.3	0.1	16.2	0.1	6.7
Part-time work, early family^c^	Employed full-time until early 20s; part-time employed from early 20s; marriage and children from early 20s	0.3	21.4	0.3	18.1	0.2	12.9
No paid work, early family^c^	Employed part-time until early 20s; homemaking from early 20s; marriage and children from early 20s	0	12.5	0.1	4.1	0.2	3.2

Abbreviations: BCS, British Cohort Study (the 1970 British birth cohort); BMI, body mass index; NCDS, National Child Development Study (the 1958 British birth cohort); NSHD, MRC National Survey of Health and Development (the 1946 British birth cohort).

a Percentages are presented as data are multiply imputed.

b Descriptive results presented for those with complete work-family histories following imputation and at least one BMI measure.

c These work-family types are not considered further for men as few men occupy these groups and therefore results are unlikely to be reliable.

**Table 2:** Predicted mean BMIs at ages 16 and 42 by work-family life course type for men in three British birth cohorts

	*Predicted mean BMI at age 16*	*95% CI*	*Predicted mean BMI at age 42*	*95% CI*	*Increase in mean BMI between 16 and 42*	*95% CI*
*NSHD*						
Work, later family	19.94	19.15, 20.73	24.62	23.51, 25.72	4.68	4.36, 5.00
Work, early family	20.13	19.29, 20.97	25.46	24.28, 26.64	5.33	4.99, 5.66
Work, marriage, non-parent	20.09	19.24, 20.94	25.09	23.82, 26.36	5.00	4.58, 5.41
Work, no family	19.74	18.89, 20.59	24.95	23.66, 26.23	5.21	4.77, 5.64
Work, divorced parent	19.89	18.78, 21.00	25.75	23.94, 27.55	5.86	5.16, 6.55
Teen parent	20.30	19.16, 21.44	26.76	25.14, 28.37	6.46	5.98, 6.93
Work, cohabitation, later parent	20.07	19.07, 21.07	25.34	23.53, 27.14	5.27	4.46, 6.07
Unstable work, no family	20.77	18.96, 22.58	25.26	24.60, 27.80	4.49	5.64, 5.22

*NCDS*						
Work, later family	20.69	20.23, 21.15	26.71	26.22, 27.20	6.02	5.99, 6.05
Work, early family	20.80	20.32, 21.28	27.32	26.75, 27.89	6.52	6.44, 6.60
Work, marriage, non-parent	20.80	20.29, 21.32	27.13	26.47, 27.79	6.33	6.18, 6.48
Work, no family	20.54	20.04, 21.04	26.54	25.92, 27.16	6.00	5.88, 6.12
Work, divorced parent	20.64	20.09, 21.19	26.05	25.30, 26.80	5.41	5.21, 5.61
Teen parent	20.34	19.58, 21.10	27.76	26.44, 29.08	7.42	6.86, 7.98
Work, cohabitation, later parent	20.52	20.00, 21.04	26.76	26.08, 27.44	6.24	6.08, 6.40
Unstable work, no family	20.71	20.00, 21.42	26.26	25.07, 27.45	5.55	5.07, 6.03

*BCS*						
Work, later family	21.39	20.60, 22.18	27.68	26.87, 28.49	6.29	6.27, 6.31
Work, early family	21.46	20.61, 22.31	28.69	27.76, 29.62	7.23	7.16, 7.30
Work, marriage, non-parent	21.42	20.54, 22.30	27.85	26.88, 28.82	6.43	6.34, 6.52
Work, no family	21.28	20.45, 22.11	27.51	26.62, 28.40	6.23	6.17, 6.29
Work, divorced parent	21.29	20.28, 22.30	28.47	27.29, 29.65	7.18	7.01, 7.35
Teen parent	19.40	17.68, 21.12	26.76	24.48, 29.04	7.36	6.80, 7.92
Work, cohabitation, later parent	20.97	20.11, 21.83	27.53	26.60, 28.46	6.56	6.48, 6.64
Unstable work, no family	20.34	19.29, 21.39	27.36	26.13, 28.59	7.02	6.85, 7.19

Abbreviations: BCS, British Cohort Study; BMI, body mass index; CI, confidence interval; NCDS, National Child Development Study; NSHD, National Survey of Health and Development.

Models adjusted for birthweight, child BMI, early-life health (internalising and externalising symptoms, reported health concerns, hospital admissions), educational attainment, parental interest in the child's education, reading scores, childhood social class and household adult social class.

**Table 3:** Predicted mean BMIs at ages 16 and 42 by work-family life course type for women in three British birth cohorts

	*Predicted mean BMI at age 16*	*95% CI*	*Predicted mean BMI at age 42*	*95% CI*	*Increase in mean BMI between 16 and 42*	*95% CI*
*NSHD*						
Work, later family	20.44	19.38, 21.50	24.93	23.34, 26.52	4.49	3.96, 5.02
Work, early family	20.64	19.41, 21.87	24.70	22.59, 26.81	4.06	3.18, 4.94
Work, marriage, non-parent	20.30	19.06, 21.54	23.96	21.81, 26.11	3.66	2.75, 4.57
Work, no family	21.14	19.88, 22.40	24.25	22.07, 26.43	3.11	2.19, 4.02
Work, divorced parent	20.19	18.53, 21.85	23.29	19.83, 26.75	3.10	1.30, 4.90
Teen parent	20.58	19.28, 21.88	24.12	21.81, 26.43	3.54	2.53, 4.55
Work, cohabitation, later parent	20.89	19.32, 22.46	24.55	22.61, 26.49	3.66	3.29, 4.03
Unstable work, no family	24.08	21.66, 26.50	29.08	25.02, 33.14	5.00	3.36, 6.64
Later family, work break	20.66	19.43, 21.89	24.47	22.36, 26.57	3.81	2.93, 4.68
Early family, work break	20.39	19.16, 21.62	24.96	22.85, 27.07	4.57	3.68, 5.45
Part-time work, early family	20.55	19.32, 21.78	24.26	22.17, 26.35	3.71	2.84, 4.57
No paid work, early family	20.62	19.38, 21.86	24.97	22.82, 27.12	4.35	3.44, 5.26

*NCDS*						
Work, later family	20.82	20.28, 21.36	25.42	24.76, 26.08	4.60	4.47, 4.73
Work, early family	20.92	20.32, 21.52	26.35	25.47, 27.23	5.43	5.16, 5.70
Work, marriage, non-parent	20.49	19.88, 21.10	25.25	24.35, 26.15	4.76	4.47, 5.05
Work, no family	20.73	20.12, 21.34	24.79	23.90, 25.68	4.06	3.77, 4.35
Work, divorced parent	20.51	19.83, 21.19	24.97	23.87, 26.07	4.46	4.04, 4.88
Teen parent	20.60	19.81, 21.39	25.63	24.30, 26.96	5.03	4.49, 5.57
Work, cohabitation, later parent	20.95	20.31, 21.59	24.71	23.74, 25.68	3.76	3.43, 4.09
Unstable work, no family	21.55	20.59, 22.51	26.74	24.93, 28.55	5.19	4.34, 6.04
Later family, work break	20.69	20.09, 21.29	25.25	24.38, 26.12	4.56	4.29, 4.83
Early family, work break	20.85	20.25, 21.45	26.01	25.15, 26.87	5.16	4.90, 5.42
Part-time work, early family	20.87	20.28, 21.46	25.77	24.92, 26.62	4.90	4.64, 5.16
No paid work, early family	20.76	20.11, 21.41	26.29	25.28, 27.30	5.53	5.18, 5.88

*BCS*						
Work, later family	22.06	21.18, 22.93	26.57	25.61, 27.52	4.51	4.43, 4.59
Work, early family	22.11	21.11, 23.11	27.85	26.60, 29.10	5.74	5.49, 6.00
Work, marriage, non-parent	21.80	20.82, 22.78	27.26	26.06, 28.46	5.46	5.24, 5.68
Work, no family	21.80	20.85, 22.75	26.74	25.60, 27.88	4.94	4.75, 5.13
Work, divorced parent	21.81	20.75, 22.87	26.83	25.45, 28.21	5.02	4.70, 5.34
Teen parent	21.71	20.59, 22.83	28.02	26.57, 29.47	6.31	5.98, 6.64
Work, cohabitation, later parent	21.81	20.84, 22.78	26.36	25.18, 27.54	4.55	4.34, 4.76
Unstable work, no family	21.84	20.65, 23.03	26.85	25.24, 28.46	5.01	4.59, 5.43
Later family, work break	21.89	20.93, 22.85	26.51	25.34, 27.68	4.62	4.42, 4.82
Early family, work break	22.27	21.26, 23.28	27.69	26.44, 28.94	5.42	5.18, 5.66
Part-time work, early family	22.02	21.06, 22.98	27.16	26.00, 28.32	5.14	4.94, 5.34
No paid work, early family	21.92	20.82, 23.02	28.61	27.18, 30.04	6.69	6.36, 7.02

Abbreviations: BCS, British Cohort Study; BMI, body mass index; CI, confidence interval; NCDS, National Child Development Study; NSHD, National Survey of Health and Development.

Models adjusted for birthweight, child BMI, early-life health (internalising and externalising symptoms, reported health concerns, hospital admissions), educational attainment, parental interest in the child's education, reading scores, childhood social class and household adult social class.

## References

[bib1] Johansson G, Huang Q, Lindfors P. A life-span perspective on women's careers, health, and well-being. Soc Sci Med 2007; 65: 685–697. 17493728 10.1016/j.socscimed.2007.04.001

[bib2] Hewitt B, Baxter J, Western M. Family, work and health: the impact of marriage, parenthood and employment on self-reported health of Australian men and women. J Sociol 2006; 42: 61–78.

[bib3] McMunn A, Bartley M, Hardy R, Kuh D. Life course social roles and women's health in mid-life: causation or selection? J Epidemiol Community Health 2006; 60: 484–489. 16698977 10.1136/jech.2005.042473 PMC2563934

[bib4] McMunn A, Bartley M, Kuh D. Women's health in mid-life: life course social roles and agency as quality. Soc Sci Med 2006; 63: 1561–1572. 16698159 10.1016/j.socscimed.2006.03.039

[bib5] Janzen B, Muhajarine N. Social role occupancy, gender, income adequacy, life stage and health: a longitudinal study of employed Canadian men and women. Soc Sci Med 2003; 57: 1491–1503. 12927478 10.1016/s0277-9536(02)00544-0

[bib6] Sabbath EL, Guevara IM, Glymour MM, Berkman LF. Use of life course work-family profiles to predict mortality risk among US women. Am J Public Health 2015; 105: e96–e102. 25713976 10.2105/AJPH.2014.302471 PMC4358181

[bib7] Lacey RE, Sacker A, Kumari M, Worts D, McDonough P, Booker CL et al. Work-family life courses and markers of stress and inflammation in mid-life: evidence from the National Child Development Study. Int J Epidemiol 2016; 45: 1247–1259. 26467761 10.1093/ije/dyv205 PMC5841625

[bib8] McMunn A, Lacey RE, Kumari M, Worts D, McDonough P, Sacker A. Work-family life courses and metabolic markers in mid-life: evidence from the British National Child Development Study. J Epidemiol Community Heal 2016; 70: 481–487. 10.1136/jech-2015-206036 PMC4853544 26659761

[bib9] Nordenmark M. Multiple social roles and well-being: a longitudinal test of the role stress theory and the role expansion theory. Acta Sociol 2004; 47: 115–126.

[bib10] Pailhe A, Robette N, Solaz A. Work and family over the life course: a typology of French long-lasting couples using optimal matching. Longit Life Course Stud 2013; 4: 196–217.

[bib11] Schober P. The parenthood effect on gender inequality: explaining the change in paid and domestic work when British couples become parents. Eur Sociol Rev 2013; 29: 74–85.

[bib12] Einiö E, Nisén J, Martikainen P. Is young fatherhood causally related to midlife mortality? A sibling fixed-effect study in Finland. J Epidemiol Community Health 2015; 69: 1077–1082. 26240236 10.1136/jech-2015-205627

[bib13] Hardy R, Lawlor DA, Black S, Mishra GD, Kuh D. Age at birth of first child and coronary heart disease risk factors at age 53 years in men and women: British birth cohort study. J Epidemiol Community Health 2009; 63: 99–105. 18782806 10.1136/jech.2008.076943 PMC2613438

[bib14] Kostiainen E, Martelin T, Kestila L, Martikainen P, Koskinen S. Employee, partner, and mother: woman's three roles and their implications for health. J Fam Issues 2009; 30: 1122–1150.

[bib15] Janicki-Deverts D, Cohen S, Matthews KA, Cullen MR. History of unemployment predicts future elevations in C-reactive protein among male participants in the Coronary Artery Risk Development in Young Adults (CARDIA) Study. Ann Behav Med 2008; 36: 176–185. 18784972 10.1007/s12160-008-9056-5

[bib16] McFarland M, Hayward M, Brown D. I've got you under my skin: marital biography and biological risk. J Marriage Fam 2013; 75: 363–380. 26078480 10.1111/jomf.12015 PMC4465275

[bib17] Hintikka J, Lehto SM, Niskanen L, Huotari A, Herzig K-H, Koivumaa-Honkanen H et al. Unemployment and ill health: a connection through inflammation? BMC Public Health 2009; 9: 410. 19909544 10.1186/1471-2458-9-410 PMC2780415

[bib18] Ponomarenko V. Cumulative disadvantages of non-employment and non-standard work for career patterns and subjective well-being in retirement. Adv Life Course Res 2016. e-pub ahead of print 22 June 2016.

[bib19] Grundy E, Read S. Pathways from fertility history to later life health: results from analyses of the English Longitudinal Study of Ageing. Demogr Res 2015; 32: 107–146.

[bib20] Barban N. Family trajectories and health: a life course perspective. Eur J Popul/Rev Eur Démographie 2013; 29: 357–385.

[bib21] Björntorp P. Do stress reactions cause abdominal obesity and comorbidities? Obes Rev 2001; 2: 73–86. 12119665 10.1046/j.1467-789x.2001.00027.x

[bib22] Mongraw-Chaffin ML, Peters SAE, Huxley RR, Woodward M. The sex-specific association between BMI and coronary heart disease: a systematic review and meta-analysis of 95 cohorts with 1·2 million participants. Lancet Diabetes Endocrinol 2015; 3: 437–449. 25960160 10.1016/S2213-8587(15)00086-8 PMC4470268

[bib23] Kahn SE, Hull RL, Utzschneider KM. Mechanisms linking obesity to insulin resistance and type 2 diabetes. Nature 2006; 444: 840–846. 17167471 10.1038/nature05482

[bib24] Sabbath EL, Mejía-Guevara I, Noelke C, Berkman LF. The long-term mortality impact of combined job strain and family circumstances: a life course analysis of working American mothers. Soc Sci Med 2015; 146: 111–119. 26513120 10.1016/j.socscimed.2015.10.024 PMC4657133

[bib25] Rindfuss R, Swicegood C, Rosenfeld R. Disorder in the life course: how common and does it matter? Am Sociol Rev 1987; 52: 785–801.

[bib26] McMunn A, Lacey R, Worts D, McDonough P, Stafford M, Booker CL et al. De-standardization and gender convergence in work-family life courses in Great Britain: a multi-channel sequence analysis. Adv Life Course Res 2015; 26: 60–75.

[bib27] Koropeckyj-Cox T, Pienta A, Brown T. Women of the 1950 s and the ‘normative' life course: the implications of childlessness, fertility timing, and marital status for psychological well-being in late midlife. Int J Aging Hum Dev 2007; 64: 299–330. 17703677 10.2190/8PTL-P745-58U1-3330

[bib28] Wadsworth M, Kuh D, Richards M, Hardy R. Cohort profile: the 1946 National Birth Cohort (MRC National Survey of Health and Development). Int J Epidemiol 2006; 35: 49–54. 16204333 10.1093/ije/dyi201

[bib29] Power C, Elliott J. Cohort profile: 1958 British birth cohort. Int J Epidemiol 2005; 35: 34–41. 16155052 10.1093/ije/dyi183

[bib30] Elliott BJ, Shepherd P. Cohort profile: 1970 British Birth Cohort. Int J Epidemiol 2006; 35: 836–843. 16931528 10.1093/ije/dyl174

[bib31] BRMB T. Technical report of the 1970 British Birth Cohort: age 42 survey (2012-2013). 2014 http://www.cls.ioe.ac.uk/page.aspx?&sitesectionid=1240&sitesectiontitle=Technical+reports+on+fieldwork.

[bib32] Richards M, Abbott R, Collis G, Hackett P, Hotopf M, Kuh D et al. Childhood Mental Health and Life Chances in Post-War Britain. Sainsbury Centre for Mental Health: London, 2009.

[bib33] Halpin B. Imputing Sequence Data: Extensions to Initial and Terminal Gaps, Stata's mi. University of Limerick: Limerick, 2013, http://www3.ul.ie/sociology/pubs/wp2013-01.pdf.

[bib34] StataCorp. Stata version 13.1. 2013.

[bib35] Lesnard L. seqcomp, a sequence analysis Stata plug-in. 2008 http://laurent.lesnard.free.fr/article.php3?id_article=8.

[bib36] Henretta JC. Early childbearing, marital status, and women's health and mortality after age 50. J Health Soc Behav 2007; 48: 254–266. 17982867 10.1177/002214650704800304

[bib37] Mirowsky J. Age at first birth, health, and mortality. J Health Soc Behav 2005; 46: 32–50. 15869119 10.1177/002214650504600104

[bib38] Dariotis JK, Pleck JH, Astone NM, Sonenstein FL. Pathways of early fatherhood, marriage, and employment: a latent class growth analysis. Demography 2011; 48: 593–623. 21499850 10.1007/s13524-011-0022-7

[bib39] Moffitt TE. Teen-aged mothers in contemporary Britain. J Child Psychol Psychiatry 2002; 43: 727–742. 12236608 10.1111/1469-7610.00082

[bib40] Bewley S. Which career first? BMJ 2005; 331: 588–589. 16166111 10.1136/bmj.331.7517.588 PMC1215541

[bib41] Nomaguchi KM, Bianchi SM. Exercise time: gender differences in the effects of marriage, parenthood, and employment. J Marriage Fam 2004; 66: 413–430.

[bib42] Umberson D, Crosnoe R, Reczek C. Social relationships and health behavior across life course. Annu Rev Sociol 2010; 36: 139–157. 21921974 10.1146/annurev-soc-070308-120011 PMC3171805

[bib43] Grundy EM, Tomassini C. Marital history, health and mortality among older men and women in England and Wales. BMC Public Health 2010; 10: 554. 20843303 10.1186/1471-2458-10-554 PMC2954998

[bib44] Sbarra DA. Marriage protects men from clinically meaningful elevations in C-reactive protein: results from the National Social Life, Health, and Aging Project (NSHAP). Psychosom Med 2009; 71: 828–835. 19661186 10.1097/PSY.0b013e3181b4c4f2 PMC3625249

[bib45] Williams K, Umberson D. Marital status, marital transitions, and health: a gendered life course perspective. J Health Soc Behav 2004; 45: 81–98. 15179909 10.1177/002214650404500106 PMC4061612

[bib46] Makepeace G, Dolton P, Woods L, Joshi H, Galinda-Rueda F From school to the labour market. In: Ferri E, Bynner J, Wadsworth M (eds). Changing Britain, Changing Lives. Institute of Education: London, 2003. pp 29–70.

[bib47] Abraham WT, Russell D. Missing data: a review of current methods and applications in epidemiological research. Curr Opin Psychiatry 2004; 17: 315–321.

[bib48] Ferri E, Smith K Partnerships and parenthood. In: Ferri E, Bynner J, Wadsworth MEJ (eds). Changing Britain, Changing lives. Institute of Education: London, 2003. pp 105–132.

