# Supplementary material for: Work-family life courses and BMI trajectories in three British birth cohorts
Source: Int J Obes (Lond). 2016 Nov 29;41(2):332–9. doi: 10.1038/ijo.2016.197 (PMC5309340; doi:10.1038/ijo.2016.197)
Supplement: Supplementary Information [file ijo2016197x1.docx]

**Work-family life courses and BMI trajectories in three British birth cohorts**

**Supplementary figure 1**

**
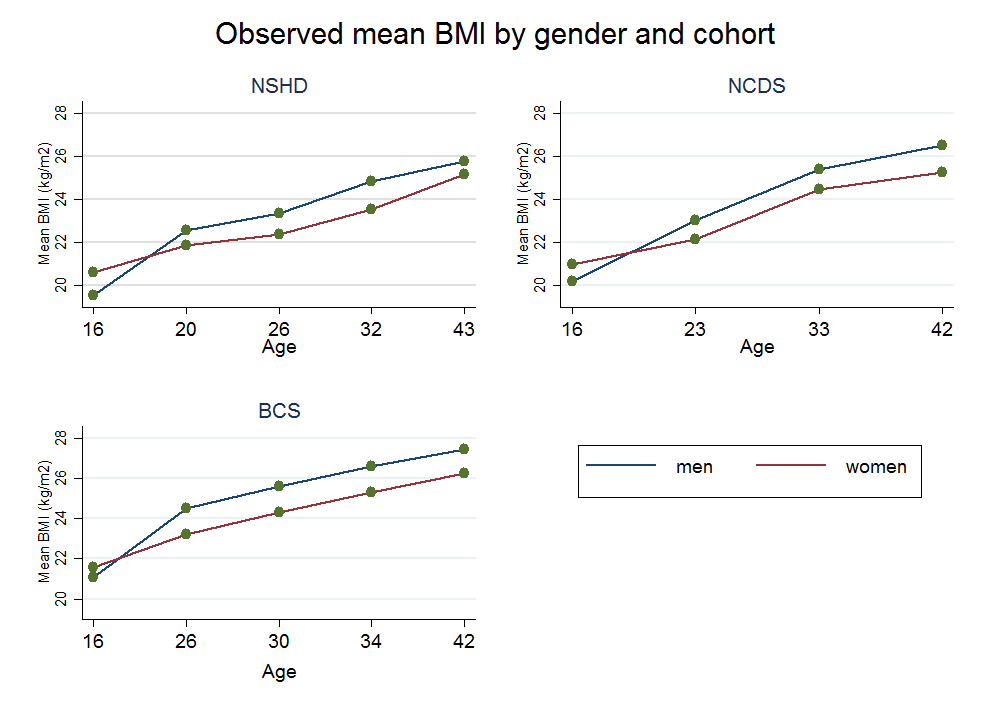
**

**Supplementary table 1.** Regression output for growth curve models of BMI trajectories by work-family type and cohort for men

|  | **NSHD** | | **NCDS** | | **BCS** | |
| --- | --- | --- | --- | --- | --- | --- |
|  | **Regression coeff** | **95% CI** | **Regression coeff** | **95% CI** | **Regression coeff** | **95% CI** |
| Intercept (42/43 yrs)^a^ | 24.60 | 23.76, 25.44 | 26.71 | 26.22, 27.20 | 27.68 | 26.87, 28.49 |
| Work-family type |  |  |  |  |  |  |
| Work, later family | Ref |  | Ref |  | Ref |  |
| Work, marriage, non-parent | 0.47 | -0.12, 1.07 | 0.42 | -0.02, 0.87 | 0.17 | -0.36, 0.71 |
| Work, no family | 0.33 | -0.28, 0.93 | -0.17 | -0.54, 0.20 | -0.17 | -0.55, 0.22 |
| Work, divorced parent | 1.13 | -0.27, 2.52 | -0.66 | -1.22, -0.10 | 0.79 | -0.07, 1.65 |
| Teen parent | 2.14 | 1.26, 3.02 | 1.05 | -0.17, 2.28 | -0.92 | -3.06, 1.23 |
| Work, cohabitation, later parent | 0.72 | -0.60, 2.04 | 0.05 | -0.43, 0.52 | -0.15 | -0.62, 0.32 |
| Unstable work, no family | 0.64 | -1.69, 2.98 | -0.45 | -1.54, 0.64 | -0.32 | -1.24, 0.61 |
| Work, early family | 0.84 | 0.46, 1.21 | 0.61 | 0.33, 0.89 | 1.01 | 0.55, 1.47 |
| Slope (time) | -0.01 | -0.03, 0.01 | 0.03 | 0.02, 0.05 | 0.06 | 0.05, 0.08 |
| Slope by work-family type interaction |  |  |  |  |  |  |
| Work, later family | Ref |  | Ref |  | Ref |  |
| Work, marriage, non-parent | 0.01 | -0.01, 0.03 | 0.02 | 0.01, 0.04 | 0.01 | -0.02, 0.03 |
| Work, no family | 0.02 | -0.005, 0.04 | -0.001 | -0.02, 0.01 | -0.002 | -0.02, 0.01 |
| Work, divorced parent | 0.04 | -0.01, 0.10 | -0.02 | -0.05, -0.001 | 0.03 | -0.002, 0.07 |
| Teen parent | 0.07 | 0.03, 0.10 | 0.05 | 0.005, 0.10 | 0.04 | -0.05, 0.13 |
| Work, cohabitation, later parent | 0.02 | -0.02, 0.07 | 0.01 | -0.01, 0.03 | 0.01 | -0.01, 0.03 |
| Unstable work, no family | -0.003 | -0.10, 0.09 | -0.02 | -0.06, 0.03 | 0.03 | -0.01, 0.07 |
| Work, early family | 0.02 | 0.01, 0.04 | 0.02 | 0.01, 0.03 | 0.04 | 0.02, 0.06 |
| Slope^2^ | -0.007 | -0.008, -0.006 | -0.008 | -0.01, -0.01 | -0.01 | -0.01, -0.01 |
| Variance in the slope | 0.01 | 0.01, 0.01 | 0.01 | 0.01, 0.02 | 0.02 | 0.02, 0.02 |
| Variance in the intercept | 9.30 | 8.44, 10.24 | 12.67 | 12.04, 13.33 | 17.16 | 16.27, 18.11 |
| Covariance of slope and intercept | 0.28 | 0.25, 0.31 | 0.41 | 0.39, 0.44 | 0.47 | 0.43, 0.50 |

Models adjusted for birthweight, child BMI, early-life health (internalising and externalising symptoms, reported health concerns, hospital admissions), educational attainment, parental interest in the child’s education, reading scores, childhood social class and household adult social class

^a^Intercept set at age 43 for the NSHD and model estimates used to calculate mean BMI at age 42

**Supplementary table 2.** Regression output for growth curve models of BMI trajectories by work-family type and cohort for women

|  | **NSHD** | | **NCDS** | | **BCS** | |
| --- | --- | --- | --- | --- | --- | --- |
|  | **Regression coeff** | **95% CI** | **Regression coeff** | **95% CI** | **Regression coeff** | **95% CI** |
| Intercept (42/43 yrs)^a^ | 25.13 | 23.54, 26.71 | 25.42 | 24.75, 26.08 | 26.57 | 25.62, 27.53 |
| Work-family type |  |  |  |  |  |  |
| Work, later family | Ref |  | Ref |  | Ref |  |
| Work, marriage, non-parent | -0.97 | -2.42, 0.48 | -0.17 | -0.78, 0.44 | 0.69 | -0.05, 1.42 |
| Work, no family | -0.68 | -2.17, 0.81 | -0.63 | -1.23, -0.04 | 0.17 | -0.45, 0.78 |
| Later family, work break | -0.46 | -1.84, 0.92 | -0.17 | -0.73, 0.39 | -0.06 | -0.73, 0.62 |
| Early family, work break | 0.03 | -1.37, 1.42 | 0.59 | 0.05, 1.13 | 1.12 | 0.31, 1.93 |
| Part-time work, early family | -0.67 | -2.03, 0.70 | 0.35 | -0.18, 0.88 | 0.59 | -0.08, 1.26 |
| No paid work, early family | 0.04 | -1.41, 1.50 | 0.87 | 0.11, 1.63 | 2.04 | 0.98, 3.10 |
| Work, divorced parent | -1.64 | -4.71, 1.43 | -0.45 | -1.33, 0.43 | 0.26 | -0.74, 1.26 |
| Teen parent | -0.81 | -2.48, 0.87 | 0.21 | -0.94, 1.36 | 1.45 | 0.36, 2.55 |
| Work, cohabitation, later parent | -0.38 | -2.32, 1.55 | -0.71 | -1.42, 0.01 | -0.21 | -0.90, 0.49 |
| Unstable work, no family | 4.15 | 0.41, 7.89 | 1.32 | -0.36, 3.00 | 0.28 | -1.02, 1.59 |
| Work, early family | -0.23 | -1.62, 1.16 | 0.93 | 0.36, 1.50 | 1.28 | 0.47, 2.09 |
| Slope (time) | 0.20 | 0.14, 0.27 | 0.09 | 0.07, 0.11 | 0.16 | 0.13, 0.18 |
| Slope by work-family type interaction |  |  |  |  |  |  |
| Work, later family | Ref |  | Ref |  | Ref |  |
| Work, marriage, non-parent | -0.03 | -0.09, 0.03 | 0.004 | -0.02, 0.03 | 0.04 | 0.01, 0.06 |
| Work, no family | -0.05 | -0.11, 0.01 | -0.02 | -0.05, -0.001 | 0.02 | -0.01, 0.04 |
| Later family, work break | -0.02 | -0.08, 0.04 | -0.003 | -0.02, 0.02 | 0.004 | -0.02, 0.03 |
| Early family, work break | 0.003 | -0.06, 0.06 | 0.02 | 0.002, 0.04 | 0.03 | 0.003, 0.07 |
| Part-time work, early family | -0.03 | -0.09, 0.03 | 0.01 | -0.01, 0.03 | 0.02 | -0.002, 0.05 |
| No paid work, early family | -0.005 | -0.07, 0.06 | 0.03 | 0.001, 0.06 | 0.08 | 0.04, 0.13 |
| Work, divorced parent | -0.05 | -0.17, 0.07 | -0.01 | -0.04, 0.02 | 0.02 | -0.02, 0.06 |
| Teen parent | -0.03 | -0.10, 0.04 | 0.01 | -0.03, 0.05 | 0.07 | 0.02, 0.11 |
| Work, cohabitation, later parent | -0.03 | -0.11, 0.05 | -0.03 | -0.06, -0.002 | 0.002 | -0.03, 0.03 |
| Unstable work, no family | 0.02 | -0.08, 0.11 | 0.03 | -0.04, 0.09 | 0.02 | -0.03, 0.07 |
| Work, early family | -0.02 | -0.07, 0.04 | 0.03 | 0.01, 0.05 | 0.05 | 0.02, 0.08 |
| Slope^2^ | 0.001 | 0.0004, 0.002 | -0.003 | -0.004, -0.003 | -0.001 | -0.001, -0.0002 |
| Variance in the slope | 0.02 | 0.02, 0.02 | 0.02 | 0.02, 0.02 | 0.03 | 0.03, 0.03 |
| Variance in the intercept | 16.03 | 13.99, 18.37 | 17.41 | 16.60, 18.25 | 26.99 | 25.71, 28.33 |
| Covariance of slope and intercept | 0.52 | 0.45, 0.60 | 0.53 | 0.50, 0.56 | 0.78 | 0.73, 0.82 |

Models adjusted for birthweight, child BMI, early-life health (internalising and externalising symptoms, reported health concerns, hospital admissions), educational attainment, parental interest in the child’s education, reading scores, childhood social class and household adult social class

^a^Intercept set at age 43 for the NSHD and model estimates used to calculate mean BMI at age 42

**Supplementary table 3.** Descriptives of all analysis variable by work-family type and cohort for men

|  | **NSHD** | | | | | | | | | |
| --- | --- | --- | --- | --- | --- | --- | --- | --- | --- | --- |
|  | **All men**  **%** | **Work, later family**  **%** | **Work, early family**  **%** | | **Work, marriage, non-parent**  **%** | **Work, no family**  **%** | **Work, divorced parent**  **%** | **Teen parent**  **%** | **Work, cohabitation, later parent**  **%** | **Unstable work, no family**  **%** |
| Birthweight (kg), mean (SD) | 3.5  (0.5) | 3.5  (0.5) | 3.5  (0.5) | | 3.5  (0.5) | 3.5  (0.5) | 3.8  (0.5) | 3.2  (0.5) | 3.5  (0.4) | 3.0  (0.5) |
| Child BMI (11 yrs), mean (SD) | 17.3  (2.1) | 17.3  (2.3) | 17.3  (2.0) | | 17.4  (2.4) | 16.9  (1.9) | 16.9  (1.5) | 15.4  (1.0) | 17.3  (2.3) | 16.0  (2.1) |
| Childhood social class (4-15 yrs) |  |  |  | |  |  |  |  |  |  |
| I | 3.3 | 3.7 | 1.8 | | 7.0 | 7.3 | 0 | 0 | 2.6 | 0 |
| II | 14.7 | 16.3 | 15.2 | | 11.4 | 10.8 | 10.3 | 0 | 9.1 | 30.4 |
| IIINM | 8.2 | 11.1 | 6.6 | | 9.5 | 7.0 | 15.4 | 0 | 6.5 | 6.5 |
| IIIM | 46.7 | 45.5 | 49.4 | | 42.9 | 41.1 | 53.9 | 66.7 | 42.9 | 21.7 |
| IV | 18.3 | 14.2 | 19.2 | | 21.3 | 22.0 | 5.1 | 33.3 | 28.6 | 15.2 |
| V | 8.9 | 9.2 | 7.8 | | 7.9 | 11.8 | 15.4 | 0 | 10.4 | 26.1 |
| Internalising disorders (13/15 yrs) |  |  |  | |  |  |  |  |  |  |
| Absent | 58.4 | 56.0 | 63.0 | | 58.1 | 48.8 | 66.1 | 5.6 | 48.2 | 50.3 |
| Mild | 32.8 | 34.5 | 30.0 | | 32.2 | 38.6 | 22.8 | 88.9 | 45.7 | 18.0 |
| Severe | 8.9 | 9.5 | 7.0 | | 9.7 | 12.7 | 11.1 | 5.6 | 6.2 | 31.7 |
| Externalising disorders (13/15 yrs) |  |  |  | |  |  |  |  |  |  |
| Absent | 71.3 | 76.1 | 67.9 | | 79.7 | 75.7 | 63.7 | 50.0 | 61.7 | 40.6 |
| Mild | 19.8 | 16.0 | 22.0 | | 16.3 | 15.8 | 33.9 | 50.0 | 18.5 | 41.8 |
| Severe | 8.9 | 8.0 | 10.2 | | 4.0 | 8.5 | 2.3 | 0 | 19.8 | 17.6 |
| Health problems (15 yrs) |  |  |  | |  |  |  |  |  |  |
| No | 91.2 | 93.3 | 91.1 | | 90.7 | 88.4 | 85.0 | 77.7 | 93.1 | 81.3 |
| Yes | 8.8 | 6.7 | 8.8 | | 9.3 | 11.6 | 15.0 | 22.3 | 6.9 | 18.8 |
| Hospital admissions (11 yrs) |  |  |  | |  |  |  |  |  |  |
| Never | 83.7 | 87.2 | 84.8 | | 83.3 | 81.4 | 83.7 | 100.0 | 84.0 | 17.4 |
| Yes | 16.3 | 12.8 | 15.2 | | 16.7 | 18.6 | 16.3 | 0 | 16.1 | 82.6 |
| Reading comprehension (11 yrs), mean (SD) | 35.2 (11.1) | 36.4  (10.7) | 35.0  (10.9) | | 37.5  (9.0) | 33.4  (12.9) | 33.4  (12.0) | 32.1  (6.6) | 32.6  (12.1) | 29.8  (12.4) |
| Parental interest in child’s education (11 yrs) |  |  |  | |  |  |  |  |  |  |
| Little or no interest | 18.6 | 13.1 | 21.6 | | 17.6 | 19.4 | 17.1 | 5.1 | 20.1 | 29.1 |
| Average interest | 48.6 | 49.6 | 49.0 | | 50.5 | 40.3 | 61.2 | 69.7 | 48.4 | 31.2 |
| Much interest | 32.8 | 37.3 | 29.4 | | 31.8 | 40.3 | 21.7 | 25.2 | 31.5 | 39.7 |
| Educational attainment (26 yrs) |  |  |  | |  |  |  |  |  |  |
| No qualifications | 42.2 | 32.3 | 47.8 | | 31.7 | 47.3 | 55.8 | 27.8 | 48.2 | 47.4 |
| CSE/O-level | 22.2 | 21.6 | 24.0 | | 19.9 | 19.4 | 16.3 | 27.8 | 13.6 | 28.1 |
| A-level | 25.9 | 33.2 | 21.2 | | 34.4 | 21.9 | 20.9 | 44.4 | 28.4 | 23.0 |
| Higher qualification/degree | 9.7 | 12.9 | 6.9 | | 14.1 | 11.4 | 7.0 | 0 | 9.9 | 1.6 |
| Household social class (15-43 yrs) |  |  |  | |  |  |  |  |  |  |
| I | 6.7 | 8.8 | 4.7 | | 12.4 | 6.9 | 3.1 | 2.2 | 4.7 | 2.5 |
| II | 24.4 | 26.6 | 24.9 | | 25.5 | 18.0 | 30.9 | 13.3 | 27.6 | 11.7 |
| IIINM | 12.2 | 14.5 | 10.9 | | 11.9 | 13.3 | 11.0 | 1.1 | 13.4 | 11.2 |
| IIIM | 33.6 | 30.8 | 36.3 | | 30.7 | 30.5 | 39.8 | 56.7 | 31.5 | 18.1 |
| IV | 12.5 | 9.5 | 14.2 | | 8.9 | 15.5 | 6.8 | 26.7 | 15.7 | 10.7 |
| V | 3.4 | 3.2 | 3.4 | | 2.8 | 4.5 | 5.8 | 0 | 2.2 | 7.3 |
| not working | 7.1 | 6.6 | 5.7 | | 7.8 | 11.4 | 2.6 | 0 | 4.9 | 38.5 |
|  | **NCDS** | | | | | | | | | |
|  | **All men**  **%** | **Work, later family**  **%** | | **Work, early family**  **%** | **Work, marriage, non-parent**  **%** | **Work, no family**  **%** | **Work, divorced parent**  **%** | **Teen parent**  **%** | **Work, cohabitation, later parent**  **%** | **Unstable work, no family**  **%** |
| Birthweight (kg), mean(SD) | 3.4 (0.5) | 3.4 (0.5) | | 3.4 (0.5) | 3.5 (5.0) | 3.4 (0.5) | 3.4 (0.5) | 3.3 (0.5) | 3.4 (0.5) | 3.2 (0.6) |
| Child BMI (11 yrs), mean (SD) | 17.3 (2.4) | 17.2 (2.3) | | 17.3 (2.4) | 17.4 (2.6) | 17.1 (2.4) | 17.1 (2.3) | 17.5 (2.4) | 17.3 (2.3) | 17.1 (2.8) |
| Childhood social class (7-16 yrs) |  |  | |  |  |  |  |  |  |  |
| I | 6.1 | 8.2 | | 3.2 | 5.0 | 9.0 | 4.3 | 2.3 | 5.7 | 11.0 |
| II | 16.0 | 19.8 | | 12.5 | 14.5 | 20.0 | 10.9 | 14.1 | 13.1 | 9.7 |
| IIINM | 10.7 | 11.9 | | 8.2 | 14.7 | 12.4 | 7.7 | 9.7 | 10.2 | 9.9 |
| IIIM | 43.8 | 41.0 | | 47.8 | 44.7 | 39.8 | 45.3 | 43.1 | 44.8 | 46.6 |
| IV | 17.8 | 15.0 | | 21.0 | 15.4 | 14.3 | 23.4 | 30.5 | 19.5 | 13.6 |
| V | 5.7 | 4.1 | | 7.2 | 5.8 | 4.5 | 8.4 | 0.3 | 6.8 | 9.2 |
| Internalising disorders (16 yrs) |  |  | |  |  |  |  |  |  |  |
| Absent | 52.3 | 54.7 | | 52.4 | 51.2 | 50.2 | 50.6 | 49.4 | 48.9 | 40.0 |
| Mild | 36.1 | 35.5 | | 35.7 | 37.6 | 36.3 | 37.0 | 31.2 | 38.8 | 34.8 |
| Severe | 11.7 | 9.9 | | 12.0 | 11.2 | 13.5 | 12.3 | 19.4 | 12.4 | 25.2 |
| Externalising disorders (16 yrs) |  |  | |  |  |  |  |  |  |  |
| Absent | 70.0 | 74.2 | | 65.8 | 76.8 | 75.8 | 58.0 | 75.3 | 61.1 | 53.5 |
| Mild | 20.8 | 19.3 | | 22.7 | 17.3 | 17.1 | 25.3 | 18.5 | 27.0 | 28.0 |
| Severe | 9.2 | 6.5 | | 11.5 | 5.9 | 7.0 | 16.7 | 6.3 | 12.0 | 18.5 |
| Health problems (16 yrs) |  |  | |  |  |  |  |  |  |  |
| No | 82.0 | 84.5 | | 81.7 | 82.9 | 79.6 | 78.7 | 79.8 | 81.5 | 64.5 |
| Yes | 18.0 | 15.5 | | 18.3 | 17.1 | 20.4 | 21.3 | 20.2 | 18.5 | 35.6 |
| Hospital admissions (11 yrs) |  |  | |  |  |  |  |  |  |  |
| Never | 51.3 | 52.0 | | 49.9 | 53.1 | 51.3 | 54.2 | 46.6 | 51.8 | 45.1 |
| Yes | 48.7 | 48.0 | | 50.1 | 46.9 | 48.7 | 45.8 | 53.4 | 48.2 | 54.9 |
| Reading comprehension (11 yrs), mean (SD) | 16.8 (6.2) | 17.6 (6.3) | | 15.5 (5.7) | 18.0 (6.2) | 17.9 (6.2) | 14.9 (6.1) | 17.6 (5.9) | 16.9 (6.3) | 14.8 (7.6) |
| Parental interest in child’s education (11 yrs) |  |  | |  |  |  |  |  |  |  |
| Little or no interest | 13.8 | 11.2 | | 17.3 | 8.2 | 9.2 | 22.5 | 21.6 | 15.7 | 21.4 |
| Average interest | 39.0 | 36.7 | | 43.6 | 37.3 | 33.8 | 38.6 | 33.7 | 40.6 | 40.7 |
| Much interest | 47.2 | 52.1 | | 39.1 | 54.6 | 57.0 | 38.9 | 44.7 | 43.7 | 37.9 |
| Educational attainment (23 yrs) |  |  | |  |  |  |  |  |  |  |
| No qualifications | 10.9 | 7.0 | | 14.9 | 6.6 | 6.7 | 18.0 | 13.4 | 15.5 | 25.6 |
| CSE/O-level | 46.0 | 41.4 | | 53.1 | 42.2 | 42.0 | 50.3 | 50.7 | 44.6 | 39.2 |
| A-level | 23.0 | 26.2 | | 19.6 | 27.1 | 25.1 | 19.5 | 19.2 | 18.5 | 18.3 |
| Higher qualification/degree | 20.2 | 25.5 | | 12.4 | 24.1 | 26.2 | 12.2 | 16.7 | 21.4 | 16.9 |
| Household social class (16-42 yrs) |  |  | |  |  |  |  |  |  |  |
| I | 6.9 | 9.1 | | 4.2 | 9.3 | 8.9 | 2.6 | 5.2 | 5.0 | 9.3 |
| II | 31.2 | 35.4 | | 27.2 | 33.5 | 34.7 | 22.9 | 29.7 | 29.4 | 18.4 |
| IIINM | 19.2 | 18.6 | | 20.6 | 23.5 | 17.6 | 17.3 | 20.1 | 15.9 | 14.5 |
| IIIM | 25.0 | 22.5 | | 28.6 | 19.5 | 20.7 | 32.8 | 25.3 | 28.5 | 27.2 |
| IV | 8.9 | 6.5 | | 10.2 | 6.5 | 9.3 | 14.2 | 11.1 | 10.4 | 15.2 |
| V | 2.4 | 1.6 | | 2.7 | 1.9 | 2.0 | 4.0 | 1.4 | 3.5 | 9.4 |
| not working | 6.5 | 6.4 | | 6.5 | 5.9 | 6.8 | 6.2 | 7.3 | 7.2 | 6.0 |
|  | **BCS** | | | | | | | | | |
|  | **All men**  **%** | **Work, later family**  **%** | | **Work, early family**  **%** | **Work, marriage, non-parent**  **%** | **Work, no family**  **%** | **Work, divorced parent**  **%** | **Teen parent**  **%** | **Work, cohabitation, later parent**  **%** | **Unstable work, no family**  **%** |
| Birthweight (kg), mean(SD) | 3.4 (0.5) | 3.4 (0.5) | | 3.4 (0.5) | 3.4 (0.5) | 3.4 (0.5) | 3.3 (0.5) | 3.3 (0.4) | 3.4 (0.5) | 3.3 (0.6) |
| Child BMI (10 yrs), mean (SD) | 16.8 (2.0) | 16.8 (2.0) | | 16.9 (1.9) | 16.7 (2.1) | 16.8 (2.1) | 16.9 (2.0) | 16.1 (1.5) | 16.6 (1.8) | 16.6 (2.0) |
| Childhood social class (5-16 yrs) |  |  | |  |  |  |  |  |  |  |
| I | 7.8 | 10.2 | | 4.2 | 7.8 | 9.4 | 2.8 | 0.9 | 5.6 | 5.9 |
| II | 23.0 | 27.1 | | 15.3 | 25.2 | 24.9 | 14.1 | 20.7 | 19.9 | 21.8 |
| IIINM | 10.8 | 11.8 | | 9.5 | 10.5 | 11.2 | 8.8 | 6.8 | 11.6 | 3.9 |
| IIIM | 42.8 | 39.4 | | 50.8 | 44.9 | 38.5 | 49.2 | 67.4 | 44.6 | 47.0 |
| IV | 11.5 | 8.0 | | 14.3 | 10.1 | 11.9 | 18.3 | 2.8 | 14.6 | 14.1 |
| V | 4.0 | 3.4 | | 6.0 | 1.6 | 4.2 | 6.8 | 1.4 | 3.8 | 7.3 |
| Internalising disorders (16 yrs) |  |  | |  |  |  |  |  |  |  |
| Absent | 46.1 | 44.9 | | 44.8 | 44.8 | 49.4 | 46.0 | 49.1 | 43.7 | 50.5 |
| Mild | 49.8 | 51.7 | | 50.8 | 50.1 | 46.3 | 48.4 | 49.4 | 52.1 | 43.9 |
| Severe | 4.1 | 3.3 | | 4.4 | 5.0 | 4.3 | 5.6 | 1.5 | 4.2 | 5.5 |
| Externalising disorders (16 yrs) |  |  | |  |  |  |  |  |  |  |
| Absent | 77.8 | 75.5 | | 78.7 | 75.5 | 76.9 | 81.8 | 85.3 | 81.6 | 85.8 |
| Mild | 16.3 | 18.0 | | 16.3 | 18.7 | 15.5 | 15.4 | 10.1 | 14.5 | 9.6 |
| Severe | 6.0 | 6.5 | | 5.0 | 5.8 | 7.6 | 2.8 | 4.6 | 3.9 | 4.6 |
| Health problems (16 yrs) |  |  | |  |  |  |  |  |  |  |
| No | 79.9 | 80.0 | | 81.5 | 83.1 | 80.0 | 79.1 | 73.8 | 79.4 | 63.6 |
| Yes | 20.1 | 20.0 | | 18.5 | 16.9 | 21.6 | 20.9 | 26.2 | 20.6 | 36.4 |
| Hospital admissions (10 yrs) |  |  | |  |  |  |  |  |  |  |
| Never | 74.5 | 75.2 | | 74.9 | 75.1 | 75.5 | 66.0 | 66.9 | 73.0 | 68.8 |
| Yes | 25.6 | 24.8 | | 25.1 | 24.9 | 24.5 | 34.0 | 33.1 | 27.0 | 31.2 |
| Reading comprehension (10 yrs), mean (SD) | 41.8 (12.2) | 43.9 (11.6) | | 39.0 (11.9) | 43.5 (11.2) | 42.3 (12.4) | 35.3 (12.3) | 34.2 (13.0) | 41.3 (11.9) | 36.4 (13.4) |
| Parental interest in child’s education (10 yrs) |  |  | |  |  |  |  |  |  |  |
| Little or no interest | 6.7 | 5.1 | | 8.0 | 5.0 | 5.4 | 14.5 | 15.0 | 8.3 | 14.1 |
| Average interest | 33.0 | 29.8 | | 39.5 | 32.4 | 30.4 | 41.2 | 39.2 | 35.5 | 35.5 |
| Much interest | 60.3 | 65.1 | | 52.5 | 62.6 | 64.2 | 44.3 | 45.8 | 56.2 | 50.4 |
| Educational attainment (26 yrs) |  |  | |  |  |  |  |  |  |  |
| No qualifications | 6.4 | 4.0 | | 8.2 | 3.8 | 5.4 | 14.3 | 13.8 | 7.8 | 18.4 |
| CSE/O-level | 60.2 | 53.2 | | 74.8 | 57.8 | 57.2 | 73.4 | 59.8 | 63.6 | 61.1 |
| A-level | 9.4 | 11.0 | | 6.0 | 10.8 | 10.3 | 6.9 | 10.6 | 8.8 | 6.7 |
| Higher qualification/degree | 24.0 | 31.9 | | 11.0 | 27.7 | 27.1 | 5.5 | 15.8 | 19.9 | 13.7 |
| Adult social class (16-42 yrs) |  |  | |  |  |  |  |  |  |  |
| I | 8.1 | 11.7 | | 4.7 | 9.5 | 8.2 | 2.5 | 1.7 | 5.9 | 4.9 |
| II | 36.8 | 43.0 | | 30.2 | 42.3 | 36.3 | 22.8 | 35.0 | 34.9 | 20.4 |
| IIINM | 14.6 | 14.2 | | 17.1 | 14.4 | 14.2 | 15.4 | 14.4 | 14.2 | 12.2 |
| IIIM | 24.8 | 20.5 | | 32.6 | 22.6 | 23.6 | 35.3 | 22.1 | 28.7 | 20.1 |
| IV | 8.2 | 5.4 | | 8.8 | 6.2 | 10.2 | 10.9 | 11.8 | 9.7 | 9.9 |
| V | 1.8 | 1.2 | | 2.3 | 1.2 | 2.0 | 3.2 | 2.2 | 1.8 | 2.3 |
| not working | 5.7 | 4.0 | | 4.3 | 3.8 | 5.6 | 10.0 | 12.8 | 4.9 | 30.3 |

Percentages are shown, unless otherwise stated, as data are multiply imputed

**Supplementary table 4.** Descriptives of all analysis variables by work-family type and cohort for women

|  | **NSHD** | | | | | | | | | | | | |
| --- | --- | --- | --- | --- | --- | --- | --- | --- | --- | --- | --- | --- | --- |
|  | **All women**  **%** | **Work later family**  **%** | **Work early family**  **%** | **Work, marriage, non-parent**  **%** | **Work, no family**  **%** | **Work, divorced parent**  **%** | **Teen parent**  **%** | **Work, cohabitation, later parent**  **%** | **Unstable work, no family**  **%** | **Later family, work break**  **%** | **Early family, work break**  **%** | **Part-time work, early family**  **%** | **No paid work, early family**  **%** |
| Birthweight (kg), mean (SD) | 3.3 (0.5) | 3.4 (0.5) | 3.3 (0.5) | 3.3 (0.4) | 3.3 (0.5) | 3.6 (0.5) | 3.3 (0.5) | 3.3 (0.5) | 3.0 (0.4) | 3.3 (0.5) | 3.3 (0.5) | 3.4 (0.5) | 3.3 (0.5) |
| Child BMI (11 yrs), mean (SD) | 17.5 (2.5) | 17.4 (2.6) | 17.5 (2.4) | 17.5 (2.6) | 17.6 (2.7) | 17.7 (1.6) | 17.6 (2.7) | 16.8 (1.7) | 19.6 (3.3) | 17.3 (2.3) | 17.4 (2.5) | 17.6 (2.4) | 17.5 (2.4) |
| Childhood social class (4-15 yrs) |  |  |  |  |  |  |  |  |  |  |  |  |  |
| I | 3.0 | 5.7 | 1.4 | 7.0 | 5.2 | 0 | 1.0 | 2.6 | 13.6 | 4.2 | 2.2 | 2.0 | 3.2 |
| II | 13.3 | 15.9 | 7.0 | 13.6 | 27.5 | 6.7 | 17.8 | 2.6 | 13.6 | 17.9 | 15.1 | 12.0 | 10.8 |
| IIINM | 9.8 | 12.5 | 10.0 | 5.3 | 14.4 | 33.3 | 4.0 | 5.3 | 13.6 | 12.0 | 8.3 | 10.4 | 9.7 |
| IIIM | 45.9 | 38.6 | 47.7 | 51.3 | 23.5 | 53.3 | 38.6 | 57.9 | 59.1 | 40.5 | 48.8 | 49.3 | 45.6 |
| IV | 19.5 | 20.5 | 26.3 | 17.1 | 18.3 | 0 | 24.8 | 21.1 | 0 | 20.5 | 18.6 | 19.5 | 14.5 |
| V | 8.4 | 6.8 | 7.6 | 5.7 | 11.1 | 6.7 | 13.9 | 10.5 | 0 | 4.9 | 7.0 | 7.0 | 16.2 |
| Internalising disorders (13/15 yrs) |  |  |  |  |  |  |  |  |  |  |  |  |  |
| Absent | 45.4 | 39.4 | 51.9 | 48.6 | 39.7 | 61.3 | 45.0 | 24.8 | 32.2 | 34.4 | 47.0 | 49.5 | 43.2 |
| Mild | 40.1 | 42.5 | 38.0 | 33.1 | 47.0 | 33.7 | 38.7 | 50.9 | 34.7 | 44.4 | 43.3 | 37.6 | 39.0 |
| Severe | 14.5 | 18.1 | 10.1 | 18.3 | 13.3 | 5.1 | 16.2 | 24.2 | 33.2 | 21.2 | 9.7 | 13.0 | 17.8 |
| Externalising disorders (13/15 yrs) |  |  |  |  |  |  |  |  |  |  |  |  |  |
| Absent | 78.0 | 86.2 | 77.1 | 79.9 | 81.4 | 62.0 | 78.3 | 65.4 | 62.4 | 82.0 | 82.3 | 75.9 | 72.1 |
| Mild | 16.3 | 8.2 | 14.7 | 17.3 | 11.5 | 8.4 | 16.6 | 23.8 | 17.1 | 15.2 | 12.0 | 19.0 | 22.7 |
| Severe | 5.7 | 5.6 | 8.2 | 2.8 | 7.2 | 29.6 | 5.2 | 10.8 | 20.5 | 2.9 | 5.7 | 5.1 | 5.2 |
| Health problems (15 yrs) |  |  |  |  |  |  |  |  |  |  |  |  |  |
| No | 91.3 | 91.1 | 90.6 | 93.9 | 87.9 | 100.0 | 91.8 | 98.7 | 83.1 | 91.1 | 89.3 | 92.6 | 92.0 |
| Yes | 8.7 | 8.9 | 9.4 | 6.1 | 12.1 | 0.0 | 8.2 | 1.3 | 16.9 | 8.9 | 10.7 | 7.5 | 8.0 |
| Hospital admissions (11 yrs) |  |  |  |  |  |  |  |  |  |  |  |  |  |
| Never | 86.5 | 87.1 | 84.2 | 85.0 | 90.3 | 100.0 | 86.1 | 84.2 | 47.8 | 91.5 | 86.8 | 88.1 | 81.9 |
| Yes | 13.5 | 12.9 | 15.8 | 15.0 | 9.7 | 0.0 | 13.9 | 15.8 | 52.2 | 8.5 | 13.2 | 11.9 | 18.1 |
| Reading comprehension (11 yrs), mean (SD) | 36.2 (9.7) | 39.9 (8.2) | 36.6 (9.0) | 38.8 (9.4) | 37.4 (9.4) | 33.6 (9.3) | 35.0 (10.2) | 36.4 (10.8) | 36.6 (9.1) | 38.2 (8.6) | 36.5 (9.0) | 34.8 (9.7) | 33.8 (11.3) |
| Parental interest in child’s education (11 yrs) |  |  |  |  |  |  |  |  |  |  |  |  |  |
| Little or no interest | 14.4 | 10.1 | 10.5 | 9.7 | 16.8 | 47.4 | 31.2 | 5.4 | 13.8 | 9.5 | 15.0 | 13.7 | 21.5 |
| Average interest | 50.9 | 45.9 | 58.4 | 53.4 | 31.8 | 5.3 | 44.8 | 64.0 | 17.9 | 44.5 | 52.4 | 54.4 | 50.7 |
| Much interest | 34.8 | 44.0 | 31.1 | 36.9 | 51.3 | 47.4 | 24.0 | 30.6 | 68.3 | 46.0 | 32.6 | 32.0 | 27.8 |
| Educational attainment (26 yrs) |  |  |  |  |  |  |  |  |  |  |  |  |  |
| No qualifications | 45.1 | 25.5 | 46.4 | 33.9 | 23.6 | 26.3 | 57.3 | 15.2 | 47.8 | 33.7 | 45.2 | 51.2 | 61.3 |
| CSE/O-level | 35.2 | 29.8 | 31.7 | 42.1 | 41.8 | 47.4 | 36.6 | 69.0 | 17.7 | 39.0 | 38.4 | 33.2 | 27.9 |
| A-level | 16.6 | 34.9 | 20.9 | 17.0 | 24.2 | 0 | 5.4 | 15.8 | 25.3 | 24.2 | 14.5 | 13.7 | 8.6 |
| Higher qualification/degree | 3.1 | 9.7 | 1.3 | 7.0 | 10.4 | 26.3 | 0.8 | 0 | 9.3 | 3.1 | 1.9 | 2.0 | 2.2 |
| Household social class (15-43 yrs) |  |  |  |  |  |  |  |  |  |  |  |  |  |
| I | 4.3 | 6.6 | 3.0 | 6.4 | 3.9 | 2.3 | 5.0 | 1.8 | 11.7 | 5.9 | 4.1 | 4.2 | 3.1 |
| II | 20.4 | 26.6 | 20.6 | 25.5 | 30.0 | 17.7 | 19.2 | 20.9 | 10.0 | 25.2 | 19.4 | 17.9 | 15.0 |
| IIINM | 13.7 | 14.8 | 13.0 | 17.2 | 25.6 | 15.3 | 11.6 | 6.8 | 7.1 | 20.6 | 12.3 | 10.9 | 10.0 |
| IIIM | 24.3 | 17.7 | 21.8 | 25.0 | 15.9 | 15.6 | 21.1 | 31.8 | 21.2 | 21.8 | 27.5 | 27.4 | 24.5 |
| IV | 11.5 | 12.1 | 13.2 | 14.5 | 9.6 | 9.1 | 12.2 | 11.1 | 6.4 | 13.1 | 10.2 | 10.6 | 10.9 |
| V | 3.4 | 2.3 | 3.9 | 1.6 | 3.3 | 0.4 | 3.9 | 4.9 | 4.9 | 2.5 | 3.1 | 3.4 | 4.8 |
| not working | 22.4 | 19.8 | 24.5 | 9.8 | 11.8 | 39.6 | 27.0 | 22.7 | 38.7 | 10.9 | 23.6 | 25.6 | 31.7 |
|  | **NCDS** | | | | | | | | | | | | |
|  | **All women**  **%** | **Work later family**  **%** | **Work early family**  **%** | **Work, marriage, non-parent**  **%** | **Work, no family**  **%** | **Work, divorced parent**  **%** | **Teen parent**  **%** | **Work, cohabitation, later parent**  **%** | **Unstable work, no family**  **%** | **Later family, work break**  **%** | **Early family, work break**  **%** | **Part-time work, early family**  **%** | **No paid work, early family**  **%** |
| Birthweight (kg), mean(SD) | 3.3 (0.5) | 3.3 (0.5) | 3.3 (0.5) | 3.3 (0.5) | 3.3 (0.5) | 3.3 (0.5) | 3.4 (0.5) | 3.2 (0.5) | 3.2 (0.4) | 3.3 (0.5) | 3.2 (0.5) | 3.2 (0.5) | 3.2 (0.5) |
| Child BMI (11 yrs), mean (SD) | 17.6 (2.7) | 17.6 (2.6) | 17.9 (2.8) | 17.4 (2.4) | 17.8 (2.8) | 18.0 (3.1) | 17.6 (2.5) | 17.6 (2.8) | 18.3 (3.1) | 17.4 (2.6) | 17.6 (2.7) | 17.6 (2.5) | 17.4 (2.6) |
| Childhood social class (7-16 yrs) |  |  |  |  |  |  |  |  |  |  |  |  |  |
| I | 5.4 | 7.3 | 2.9 | 5.0 | 11.9 | 4.5 | 1.5 | 5.9 | 6.6 | 7.8 | 3.0 | 3.9 | 3.8 |
| II | 15.8 | 22.1 | 12.9 | 18.0 | 21.0 | 11.2 | 10.4 | 16.3 | 16.2 | 21.0 | 12.3 | 13.2 | 8.4 |
| IIINM | 10.7 | 10.5 | 8.2 | 13.4 | 11.5 | 11.9 | 6.4 | 13.2 | 16.3 | 11.6 | 10.4 | 10.7 | 5.0 |
| IIIM | 44.6 | 38.6 | 49.5 | 44.8 | 40.6 | 51.5 | 46.9 | 42.6 | 28.0 | 39.8 | 47.9 | 46.5 | 46.7 |
| IV | 17.5 | 17.3 | 18.5 | 14.7 | 11.9 | 14.2 | 19.2 | 17.8 | 29.7 | 14.3 | 20.3 | 19.7 | 24.1 |
| V | 5.9 | 4.2 | 8.1 | 4.1 | 3.2 | 6.8 | 15.6 | 4.1 | 3.2 | 5.5 | 6.1 | 6.1 | 12.1 |
| Internalising disorders (16 yrs) |  |  |  |  |  |  |  |  |  |  |  |  |  |
| Absent | 47.5 | 49.6 | 43.9 | 52.0 | 51.4 | 45.5 | 39.8 | 48.6 | 41.9 | 51.8 | 45.3 | 46.7 | 37.0 |
| Mild | 38.3 | 38.7 | 37.5 | 37.9 | 37.2 | 40.3 | 34.3 | 37.3 | 43.8 | 35.8 | 40.6 | 39.0 | 39.4 |
| Severe | 14.2 | 11.7 | 18.6 | 10.1 | 11.4 | 14.2 | 26.0 | 14.1 | 14.3 | 12.5 | 14.1 | 14.3 | 23.6 |
| Externalising disorders (16 yrs) |  |  |  |  |  |  |  |  |  |  |  |  |  |
| Absent | 79.0 | 86.7 | 75.7 | 87.0 | 85.1 | 65.8 | 61.2 | 69.5 | 62.7 | 83.7 | 79.3 | 76.9 | 65.3 |
| Mild | 15.9 | 11.3 | 17.6 | 10.5 | 12.2 | 22.8 | 24.9 | 22.7 | 23.8 | 13.5 | 15.5 | 18.0 | 22.7 |
| Severe | 5.1 | 2.0 | 6.7 | 2.5 | 2.7 | 11.4 | 14.0 | 7.8 | 13.5 | 2.8 | 5.2 | 5.1 | 12.0 |
| Health problems (16 yrs) |  |  |  |  |  |  |  |  |  |  |  |  |  |
| No | 83.7 | 83.1 | 79.8 | 84.5 | 84.6 | 83.4 | 84.9 | 85.1 | 77.4 | 84.6 | 84.1 | 85.2 | 81.2 |
| Yes | 16.3 | 16.9 | 20.2 | 15.5 | 15.4 | 16.6 | 15.2 | 14.9 | 22.6 | 15.4 | 16.0 | 14.8 | 18.8 |
| Hospital admissions (11 yrs) |  |  |  |  |  |  |  |  |  |  |  |  |  |
| Never | 59.6 | 60.2 | 59.4 | 59.3 | 63.0 | 62.3 | 60.7 | 57.3 | 64.7 | 61.4 | 59.0 | 57.9 | 55.5 |
| Yes | 40.4 | 39.8 | 40.6 | 40.7 | 37.0 | 37.7 | 39.3 | 42.7 | 35.3 | 38.6 | 41.0 | 42.1 | 44.5 |
| Reading comprehension (11 yrs), mean (SD) | 16.7 (5.7) | 18.5 (5.7) | 15.8 (5.5) | 18.4 (5.9) | 18.7 (5.5) | 15.8 (5.6) | 13.8 (5.0) | 17.5 (5.6) | 15.9 (7.3) | 17.3 (5.6) | 15.8 (5.2) | 15.7 (5.5) | 13.4 (5.8) |
| Parental interest in child’s education (11 yrs) |  |  |  |  |  |  |  |  |  |  |  |  |  |
| Little or no interest | 11.9 | 7.5 | 13.5 | 7.3 | 7.1 | 13.7 | 27.4 | 13.5 | 28.7 | 8.5 | 12.7 | 14.0 | 23.7 |
| Average interest | 37.9 | 31.4 | 41.5 | 32.7 | 29.0 | 41.7 | 38.6 | 38.8 | 25.0 | 35.3 | 44.9 | 39.9 | 44.3 |
| Much interest | 50.2 | 61.1 | 44.9 | 60.1 | 63.9 | 44.6 | 34.0 | 47.8 | 46.3 | 56.2 | 42.4 | 46.2 | 32.1 |
| Educational attainment (23 yrs) |  |  |  |  |  |  |  |  |  |  |  |  |  |
| No qualifications | 12.9 | 6.1 | 13.2 | 7.5 | 5.7 | 13.1 | 34.7 | 12.7 | 28.1 | 10.3 | 13.0 | 16.3 | 36.5 |
| CSE/O-level | 53.8 | 43.2 | 60.1 | 48.0 | 43.8 | 67.2 | 55.0 | 56.1 | 38.7 | 45.9 | 65.9 | 56.4 | 49.8 |
| A-level | 12.9 | 15.7 | 9.9 | 18.2 | 17.3 | 8.2 | 6.1 | 12.2 | 15.6 | 18.9 | 10.2 | 9.4 | 6.7 |
| Higher qualification/degree | 20.5 | 35.0 | 16.8 | 26.3 | 33.3 | 11.5 | 4.2 | 19.1 | 17.6 | 24.9 | 10.9 | 18.0 | 7.0 |
| Household social class (16-42 yrs) |  |  |  |  |  |  |  |  |  |  |  |  |  |
| I | 6.5 | 9.1 | 4.1 | 9.8 | 8.9 | 2.6 | 1.4 | 5.6 | 7.2 | 10.4 | 4.1 | 5.0 | 3.4 |
| II | 32.6 | 41.9 | 32.8 | 39.1 | 40.4 | 26.8 | 17.7 | 32.6 | 24.1 | 34.0 | 27.9 | 29.3 | 18.5 |
| IIINM | 24.1 | 21.5 | 23.4 | 24.4 | 24.1 | 27.9 | 17.5 | 23.7 | 18.0 | 22.2 | 25.9 | 26.7 | 19.0 |
| IIIM | 19.8 | 14.6 | 22.3 | 14.6 | 13.2 | 21.8 | 29.1 | 18.8 | 20.9 | 16.5 | 23.7 | 22.0 | 30.8 |
| IV | 8.8 | 5.1 | 9.1 | 5.9 | 5.4 | 12.4 | 18.2 | 10.2 | 16.5 | 8.6 | 10.1 | 8.7 | 16.1 |
| V | 1.9 | 1.1 | 2.2 | 0.8 | 1.2 | 2.1 | 6.2 | 1.6 | 1.7 | 1.7 | 1.7 | 2.6 | 4.2 |
| not working | 6.5 | 6.8 | 6.1 | 5.6 | 6.8 | 6.5 | 9.9 | 7.5 | 11.6 | 6.6 | 6.5 | 5.7 | 8.0 |
|  | **BCS** | | | | | | | | | | | | |
|  | **All women**  **%** | **Work later family**  **%** | **Work early family**  **%** | **Work, marriage, non-parent**  **%** | **Work, no family**  **%** | **Work, divorced parent**  **%** | **Teen parent**  **%** | **Work, cohabitation, later parent**  **%** | **Unstable work, no family**  **%** | **Later family, work break**  **%** | **Early family, work break**  **%** | **Part-time work, early family**  **%** | **No paid work, early family**  **%** |
| Birthweight (kg), mean (SD) | 3.3 (0.5) | 3.3 (0.5) | 3.3 (0.5) | 3.2 (0.5) | 3.3 (0.5) | 3.2 (0.5) | 3.3 (0.5) | 3.2 (0.5) | 3.2 (0.5) | 3.2 (0.5) | 3.6 (4.4) | 3.3 (0.5) | 3.2 (0.6) |
| Child BMI (10 yrs), mean (SD) | 17.0 (2.2) | 16.9 (2.1) | 17.4 (2.4) | 17.1 (2.2) | 17.1 (2.3) | 17.1 (2.3) | 17.5 (2.6) | 16.9 (2.2) | 17.3 (2.5) | 16.8 (2.1) | 17.0 (2.2) | 17.2 (2.2) | 17.0 (2.2) |
| Childhood social class (5-16 yrs) |  |  |  |  |  |  |  |  |  |  |  |  |  |
| I | 7.1 | 10.8 | 4.9 | 6.9 | 9.5 | 1.7 | 4.4 | 6.8 | 7.1 | 7.7 | 3.6 | 6.3 | 4.2 |
| II | 22.4 | 25.3 | 15.0 | 24.4 | 28.8 | 19.0 | 11.1 | 22.4 | 22.9 | 25.1 | 15.3 | 20.2 | 10.7 |
| IIINM | 10.0 | 11.0 | 9.7 | 12.0 | 10.1 | 11.5 | 5.8 | 9.9 | 7.4 | 11.2 | 7.8 | 8.7 | 8.6 |
| IIIM | 42.6 | 41.3 | 46.3 | 41.3 | 38.3 | 47.5 | 54.8 | 41.5 | 41.9 | 40.5 | 45.8 | 46.3 | 41.1 |
| IV | 13.5 | 8.6 | 16.9 | 12.7 | 10.1 | 17.3 | 15.2 | 15.2 | 12.5 | 11.6 | 20.7 | 14.4 | 23.1 |
| V | 4.5 | 2.9 | 7.2 | 2.6 | 3.1 | 2.9 | 8.8 | 4.2 | 8.2 | 3.9 | 6.9 | 4.1 | 12.4 |
| Internalising disorders (16 yrs) |  |  |  |  |  |  |  |  |  |  |  |  |  |
| Absent | 53.7 | 50.6 | 55.1 | 51.7 | 52.1 | 55.8 | 60.1 | 53.9 | 58.0 | 55.0 | 57.2 | 53.3 | 55.9 |
| Mild | 43.8 | 47.2 | 42.3 | 45.7 | 45.0 | 39.7 | 37.4 | 44.2 | 40.7 | 42.3 | 41.1 | 44.7 | 41.4 |
| Severe | 2.5 | 2.3 | 2.7 | 2.7 | 2.9 | 4.4 | 2.5 | 1.9 | 1.3 | 2.8 | 1.7 | 2.0 | 2.8 |
| Externalising disorders (16 yrs) |  |  |  |  |  |  |  |  |  |  |  |  |  |
| Absent | 72.9 | 70.3 | 75.8 | 68.9 | 72.4 | 76.7 | 86.1 | 72.9 | 78.9 | 67.2 | 75.2 | 75.5 | 75.9 |
| Mild | 19.1 | 20.7 | 17.5 | 21.7 | 18.8 | 17.2 | 10.6 | 20.6 | 13.8 | 20.4 | 18.7 | 18.2 | 18.2 |
| Severe | 8.0 | 9.0 | 6.7 | 9.4 | 8.7 | 6.1 | 3.3 | 6.5 | 7.3 | 12.4 | 6.1 | 6.3 | 6.0 |
| Health problems (16 yrs) |  |  |  |  |  |  |  |  |  |  |  |  |  |
| No | 82.6 | 84.1 | 82.0 | 83.5 | 82.6 | 81.1 | 75.9 | 83.8 | 74.6 | 82.7 | 83.6 | 83.7 | 76.9 |
| Yes | 17.4 | 15.9 | 18.0 | 16.5 | 17.4 | 18.9 | 24.1 | 16.2 | 25.5 | 17.3 | 16.4 | 16.3 | 23.1 |
| Hospital admissions (10 yrs) |  |  |  |  |  |  |  |  |  |  |  |  |  |
| Never | 80.7 | 78.2 | 79.5 | 82.0 | 80.3 | 74.6 | 72.8 | 83.1 | 79.0 | 81.7 | 80.7 | 84.1 | 79.3 |
| Yes | 19.3 | 21.8 | 20.5 | 18.1 | 19.7 | 25.4 | 27.2 | 16.9 | 21.0 | 18.3 | 19.3 | 15.9 | 20.7 |
| Reading comprehension (10 yrs), mean (SD) | 42.8 (11.6) | 46.3 (10.4) | 41.9 (10.6) | 45.1 (10.8) | 44.4 (11.2) | 40.1 (11.9) | 36.4 (12.9) | 43.5 (11.1) | 39.6 (13.2) | 43.2 (11.7) | 39.4 (11.6) | 41.6 (11.2) | 36.1 (13.0) |
| Parental interest in child’s education (10 yrs) |  |  |  |  |  |  |  |  |  |  |  |  |  |
| Little or no interest | 6.7 | 2.8 | 6.4 | 3.4 | 3.2 | 14.7 | 18.6 | 7.3 | 14.9 | 6.0 | 8.9 | 6.2 | 23.4 |
| Average interest | 33.0 | 28.2 | 39.8 | 29.6 | 28.0 | 35.7 | 39.6 | 35.9 | 35.0 | 31.6 | 39.4 | 36.0 | 35.6 |
| Much interest | 60.3 | 69.0 | 53.8 | 67.1 | 68.9 | 49.6 | 41.8 | 56.8 | 50.1 | 62.3 | 51.7 | 57.8 | 41.0 |
| Educational attainment (26 yrs) |  |  |  |  |  |  |  |  |  |  |  |  |  |
| No qualifications | 4.7 | 1.1 | 3.2 | 2.8 | 2.5 | 6.8 | 14.4 | 4.0 | 13.3 | 4.2 | 6.5 | 6.1 | 18.4 |
| CSE/O-level | 61.6 | 49.3 | 69.4 | 53.1 | 53.0 | 71.5 | 73.8 | 63.8 | 58.6 | 60.8 | 77.8 | 70.2 | 71.1 |
| A-level | 11.0 | 13.1 | 13.1 | 14.6 | 12.9 | 9.4 | 6.0 | 10.3 | 7.6 | 10.7 | 6.0 | 9.9 | 4.5 |
| Higher qualification/degree | 22.7 | 36.6 | 14.3 | 29.5 | 31.5 | 12.3 | 5.8 | 21.9 | 20.6 | 24.4 | 9.7 | 13.9 | 6.1 |
| Household social class (16-42 yrs) |  |  |  |  |  |  |  |  |  |  |  |  |  |
| I | 6.3 | 11.3 | 4.6 | 8.2 | 8.2 | 2.7 | 2.2 | 5.3 | 4.6 | 6.5 | 3.1 | 4.0 | 2.1 |
| II | 36.1 | 45.1 | 39.5 | 46.2 | 41.4 | 29.6 | 19.1 | 38.5 | 21.9 | 32.5 | 24.4 | 32.9 | 13.2 |
| IIINM | 21.9 | 20.3 | 23.1 | 22.5 | 24.7 | 24.7 | 13.4 | 21.7 | 15.6 | 17.2 | 19.5 | 27.6 | 16.4 |
| IIIM | 16.3 | 12.6 | 16.4 | 12.6 | 12.5 | 20.4 | 22.7 | 16.8 | 16.9 | 16.7 | 23.8 | 18.3 | 24.8 |
| IV | 8.7 | 5.5 | 10.0 | 5.3 | 6.7 | 12.7 | 16.6 | 8.7 | 10.0 | 7.5 | 15.5 | 9.7 | 13.8 |
| V | 1.7 | 0.6 | 1.6 | 0.8 | 1.0 | 2.1 | 5.5 | 1.8 | 2.5 | 1.5 | 2.4 | 2.0 | 4.3 |
| not working | 9.0 | 4.7 | 4.9 | 4.4 | 5.5 | 7.8 | 20.5 | 7.3 | 28.6 | 18.1 | 11.4 | 5.4 | 25.4 |

Percentages are shown as data are multiply imputed
